# Supplementary material for: Spatially resolved free-energy contributions of native fold and molten-globule-like Crambin
Source: Biophys J. 2021 Jun 2;120(16):3470–82. doi: 10.1016/j.bpj.2021.05.019 (PMC8391029; doi:10.1016/j.bpj.2021.05.019)
Supplement: Document S1. Supporting materials and methods and Figs. S1–S7 [file mmc1.pdf]

Biophysical Journal, Volume *120*

Supplemental information

Spatially resolved free-energy contributions of native fold and molten-globule-like Crambin

Leonard P. Heinz and Helmut Grubmüller

Supplemental Information

Spatially resolved free-energy contributions of native fold and molten-globule-like Crambin

Leonard P. Heinz[1,*] and Helmut Grubmüller[1,*]

[1]Department of Theoretical and Computational Biophysics, Max-Planck Institute for Biophysical Chemistry, Göttingen, Germany

[*]Correspondence: lheinz@gwdg.de, hgrubmu@gwdg.de

UNRESTRAINED MD SIMULATION FOR THE PROTEIN ENTROPY CALCULATION

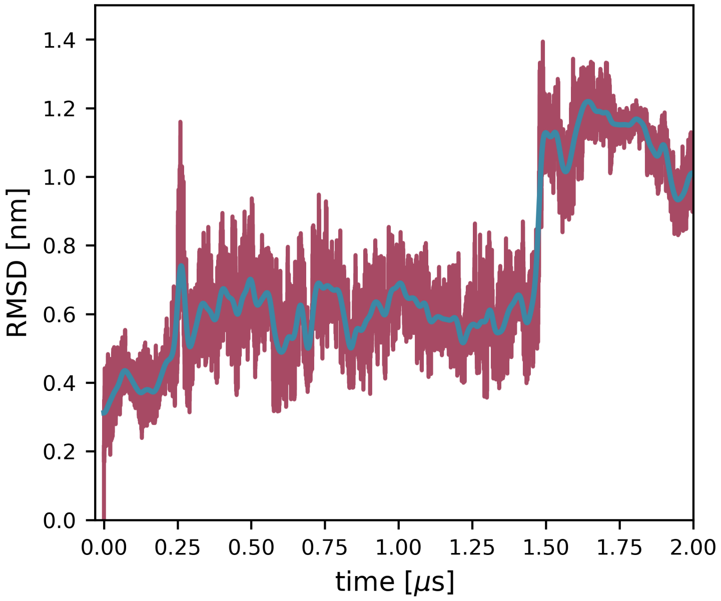

Figure S1: RMSD of the molten-globule-like conformation during an unrestrained MD simulation for the protein entropy calculation or Crambin. The 10 ns running average is shown in blue.

DECREASE OF WATER-WATER CORRELATIONS UPON BINDING TO A PROTEIN CHARGE

To show that the mutual information between a bound water molecule and a second-shell water molecule is smaller compared to bulk water, we considered two molecules with positions x and y.

In bulk, the molecules have single-body entropies $S(X)$, $S(Y)$ and the joint entropy $S(X, Y)$. Their mutual information (correlation) is $I(X, Y) = S(X) + S(Y) - S(X, Y)$.

Upon binding of the first molecule (X) to a protein charge, its entropy $\tilde{S}(X) < S(X)$ is reduced compared to bulk water

(see main text Fig. 2A). As shown by the distribution of $-T\Delta S_1$ in the main text Fig. 2A, the second-shell molecule is almost unaffected by the binding of X, i.e., its single-body entropy $\tilde{S}(Y) = S(Y)$ remains unchanged. We furthermore assume that the conditional entropy $\tilde{S}(X|Y) \leq S(X|Y)$ is also reduced compared to bulk water, as the mobility of X is hindered upon binding.

The mutual information

$$\begin{aligned}\tilde{I}(X,Y) &= \tilde{S}(X) + \tilde{S}(Y) - \tilde{S}(X,Y) \\ &= \tilde{S}(X) - \tilde{S}(X|Y) \\ &< S(X) - S(X|Y) = I(X,Y)\end{aligned}$$

is therefore smaller compared to bulk.

COMPARISON OF THE LOCAL CONVEXITY WITH THE ENTROPIC MULTI-BODY CONTRIBUTIONS

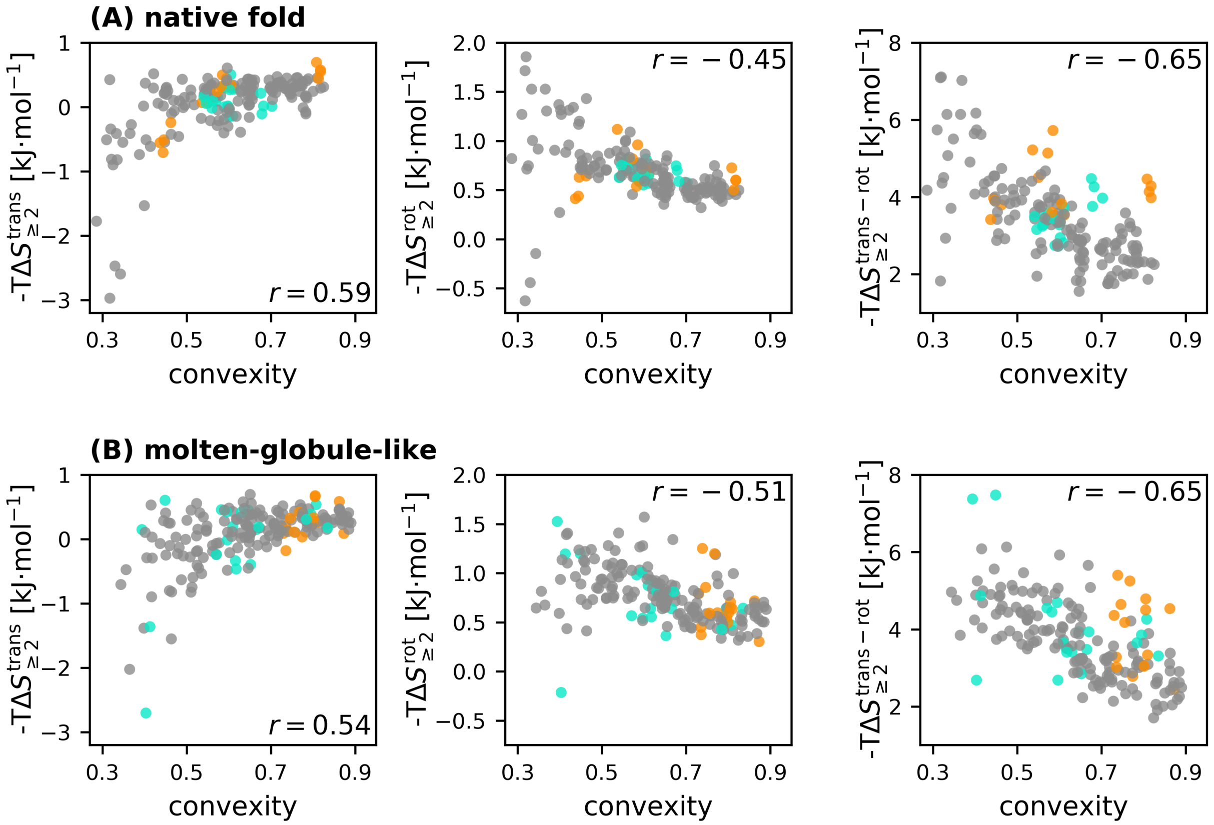

Figure S2: Dependence of the multi-body correlation contributions $-T\Delta S_{\geq 2}$ (relative to bulk water) on the local convexity for the native fold (A) and the molten-globule-like conformations (B). Charged amino acids are colored orange, polar amino acids are shown in cyan. Apolar amino acids are colored grey. Pearson correlation coefficients are stated in the corners of each plot.

ESTIMATION OF THE DENATURATION TEMPERATURES FROM THE SIMULATION RESULTS

The temperature dependence of the enthalpy difference between folded and unfolded states of a protein can be approximated as (1)

$$\Delta H(T) = \Delta H(T_c) + (T - T_c)\Delta C_P,$$

where T_c is the heat denaturation temperature and ΔC_P is the heat capacity difference between folded and unfolded states, which here is assumed to be constant. In the same manner, the entropy difference reads (1)

$$\Delta S(T) = \frac{\Delta H(T_c)}{T_c} + \Delta C_P \log\left(\frac{T}{T_c}\right).$$

Using the simulation results $\Delta H_{300} = \Delta H(300\,\mathrm{K}) \approx 228\,\mathrm{kJ{\cdot}mol^{-1}}$ and $-300\,\mathrm{K} \cdot \Delta S_{300} = -300\,\mathrm{K} \cdot \Delta S(300\,\mathrm{K}) \approx$

$-175\,\mathrm{kJ{\cdot}mol^{-1}}$, the above expressions can be rewritten as

$$\Delta H(T) = \Delta H_{300} + (T - 300\,\mathrm{K})\Delta C_P$$
$$\Delta S(T) = \Delta S_{300} + \Delta C_P \log\left(\frac{T}{300\,\mathrm{K}}\right).$$

Assuming typical values of ΔC_P between 5 and $15\,\mathrm{kJ{\cdot}mol^{-1}{\cdot}K^{-1}}$ (1, 2), the free-energy difference $\Delta G(T) = \Delta H(T) - T\Delta S(T)$ has a concave shape, as illustrated in Fig. S3.

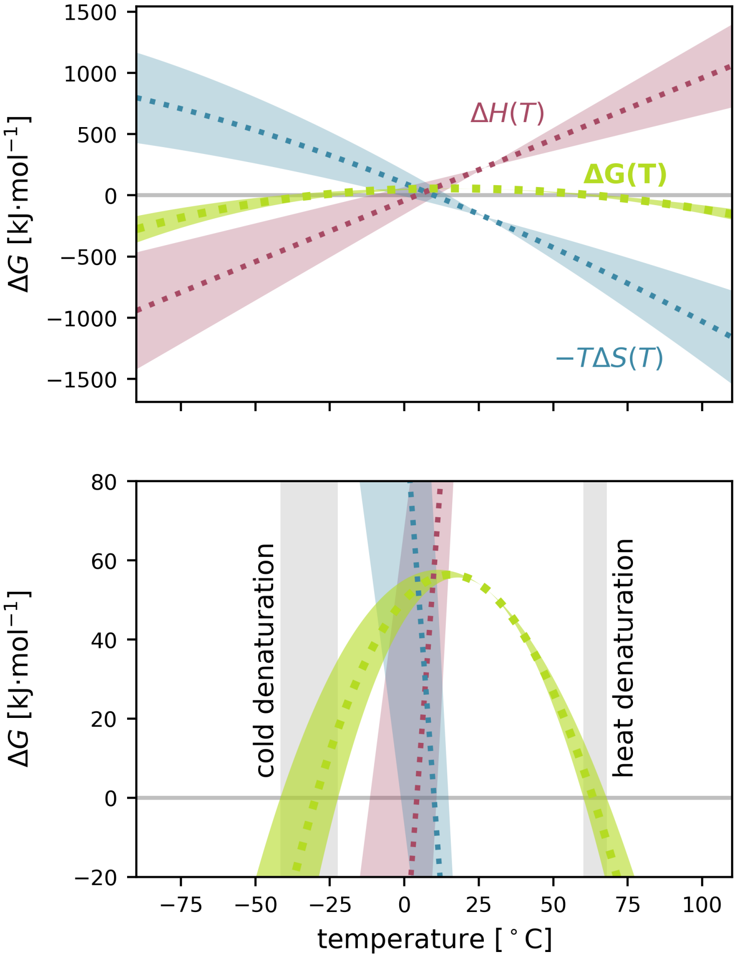

Figure S3: Temperature dependence of $\Delta H(T)$, $\Delta S(T)$, and $\Delta G(T)$ for ΔC_P between 5 and $15\,\mathrm{kJ{\cdot}mol^{-1}{\cdot}K^{-1}}$. The bottom panel is a zoomed-in version. Cold and heat denaturation temperature ranges are marked in grey.

The roots of $\Delta G(T)$, correspond to the cold (between $-42\,^\circ\mathrm{C}$ and $-22\,^\circ\mathrm{C}$) and heat denaturation (between $60\,^\circ\mathrm{C}$ and $68\,^\circ\mathrm{C}$) temperatures, respectively.

SPATIALLY RESOLVED THERMODYNAMIC QUANTITIES ON OFF-CENTER SLICES

The following figures show the spatially resolved solvent enthalpies, entropies, and free energies on slices across the three dimensional space, as visualized in Fig. 2 of the main text, but offset by 1.0, 0.5, −0.5, and −1.0 nm in depth relative to the protein center.

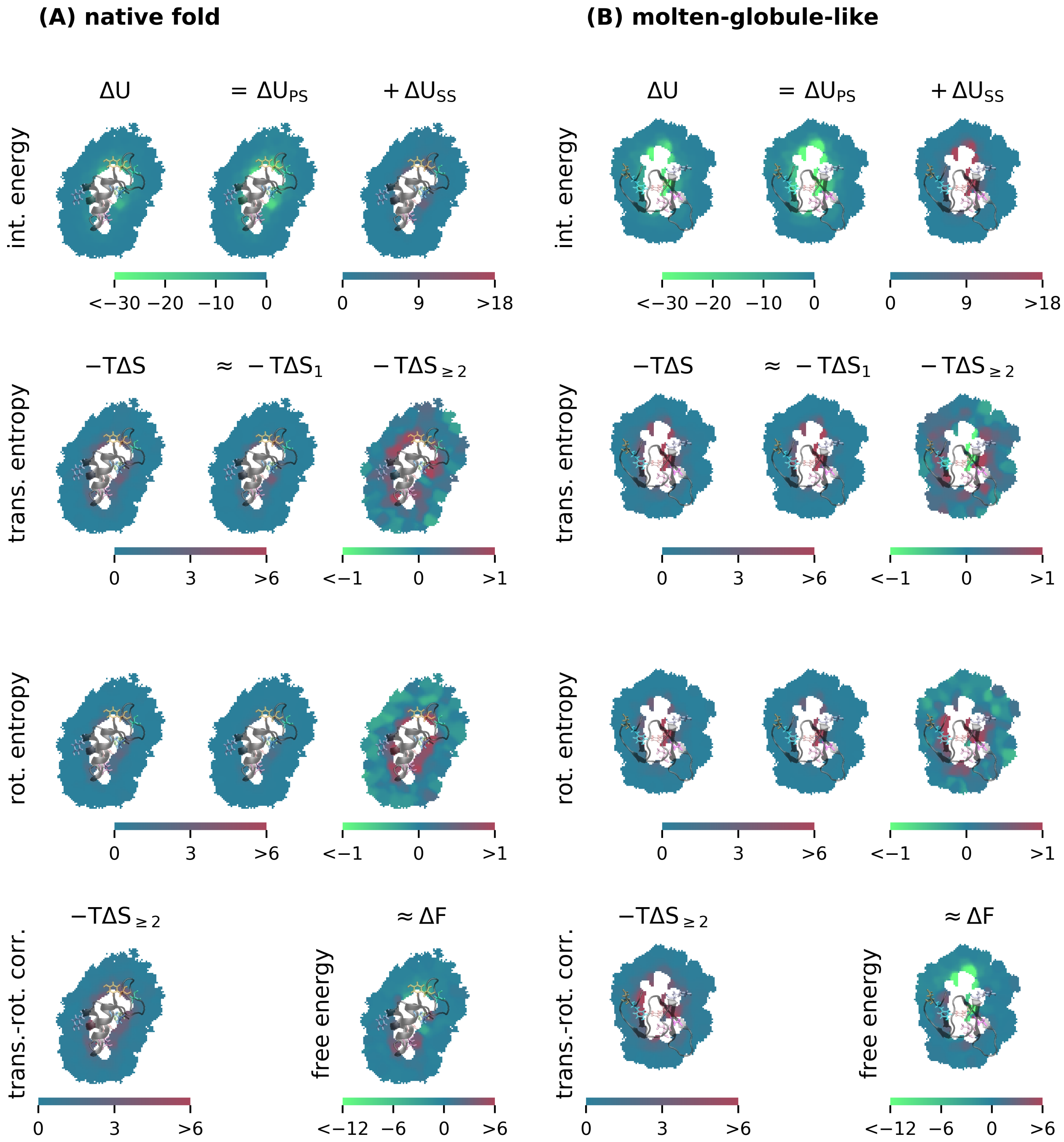

Figure S4: Spatially resolved solvent enthalpies, entropies, and free energies of Crambin in the native fold (A) and a molten-globule-like conformation (B), as shown in Fig. 2 of the main text. Visualization-slice across the three-dimensional space offset by $\approx 1.0\,\mathrm{nm}$ relative to the center of the protein.

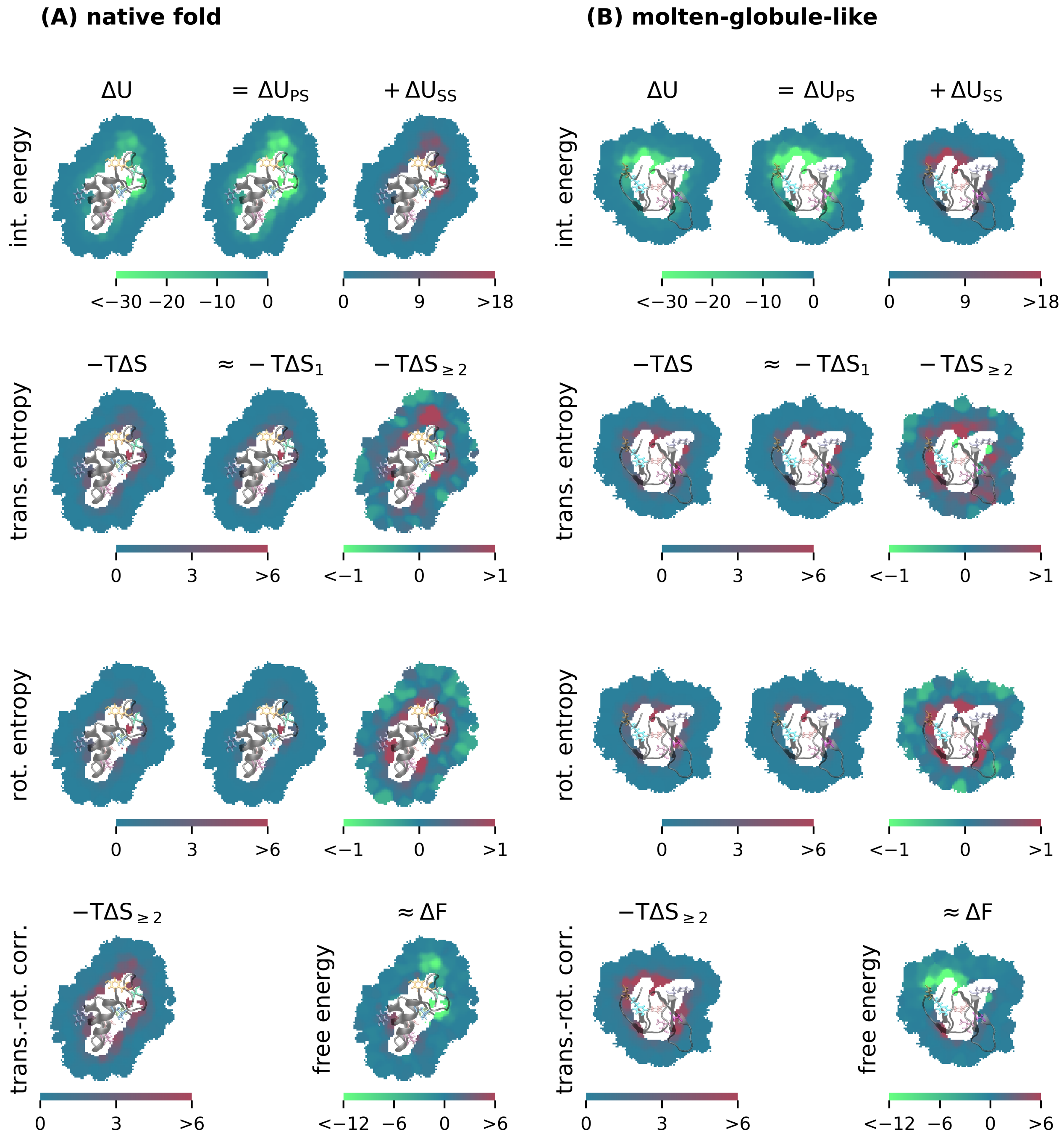

Figure S5: Spatially resolved solvent enthalpies, entropies, and free energies of Crambin in the native fold (A) and a molten-globule-like conformation (B), as shown in Fig. 2 of the main text. Visualization-slice across the three-dimensional space offset by ≈ 0.5 nm relative to the center of the protein.

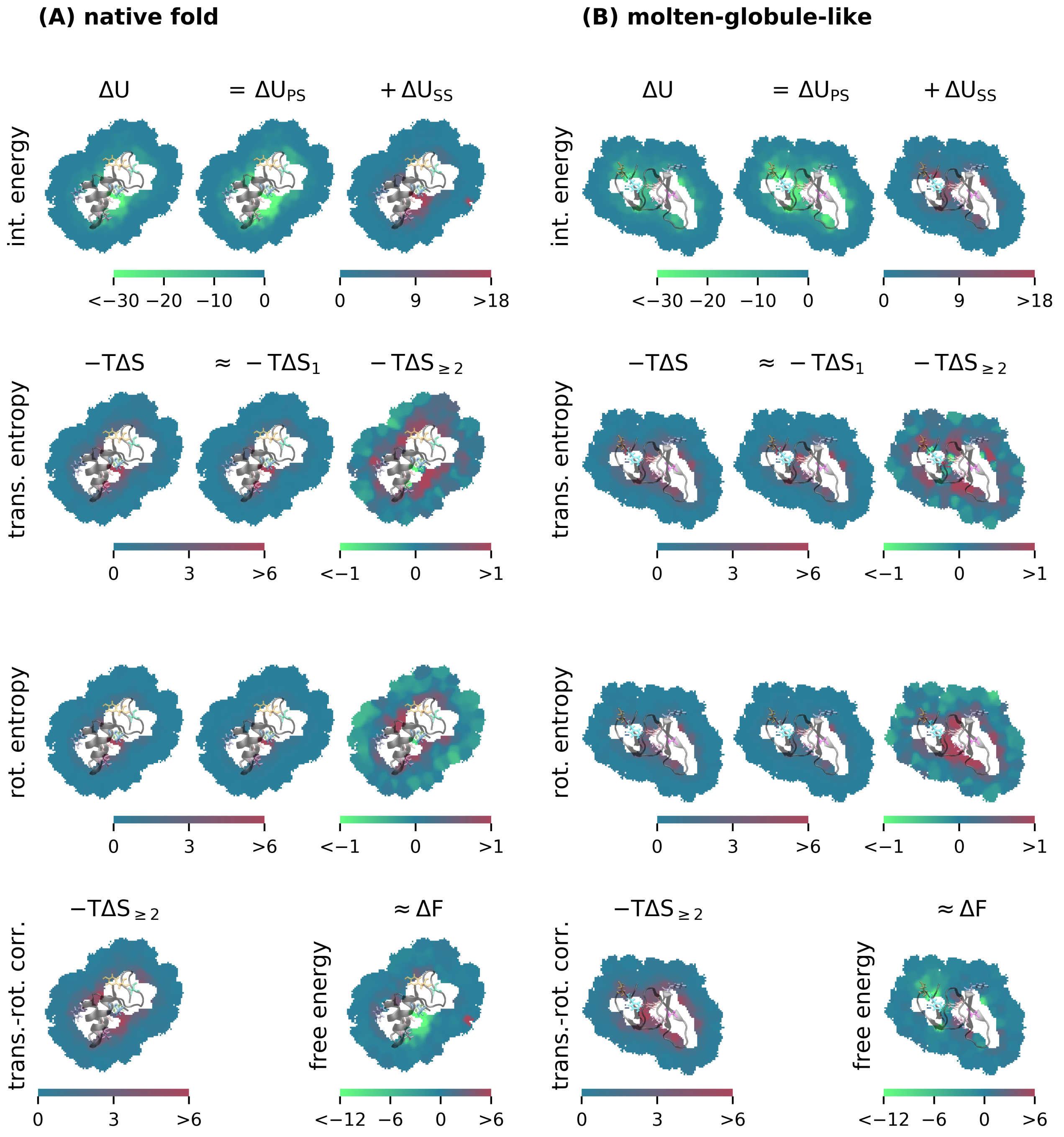

Figure S6: Spatially resolved solvent enthalpies, entropies, and free energies of Crambin in the native fold (A) and a molten-globule-like conformation (B), as shown in Fig. 2 of the main text. Visualization-slice across the three-dimensional space offset by ≈ -0.5 nm relative to the center of the protein.

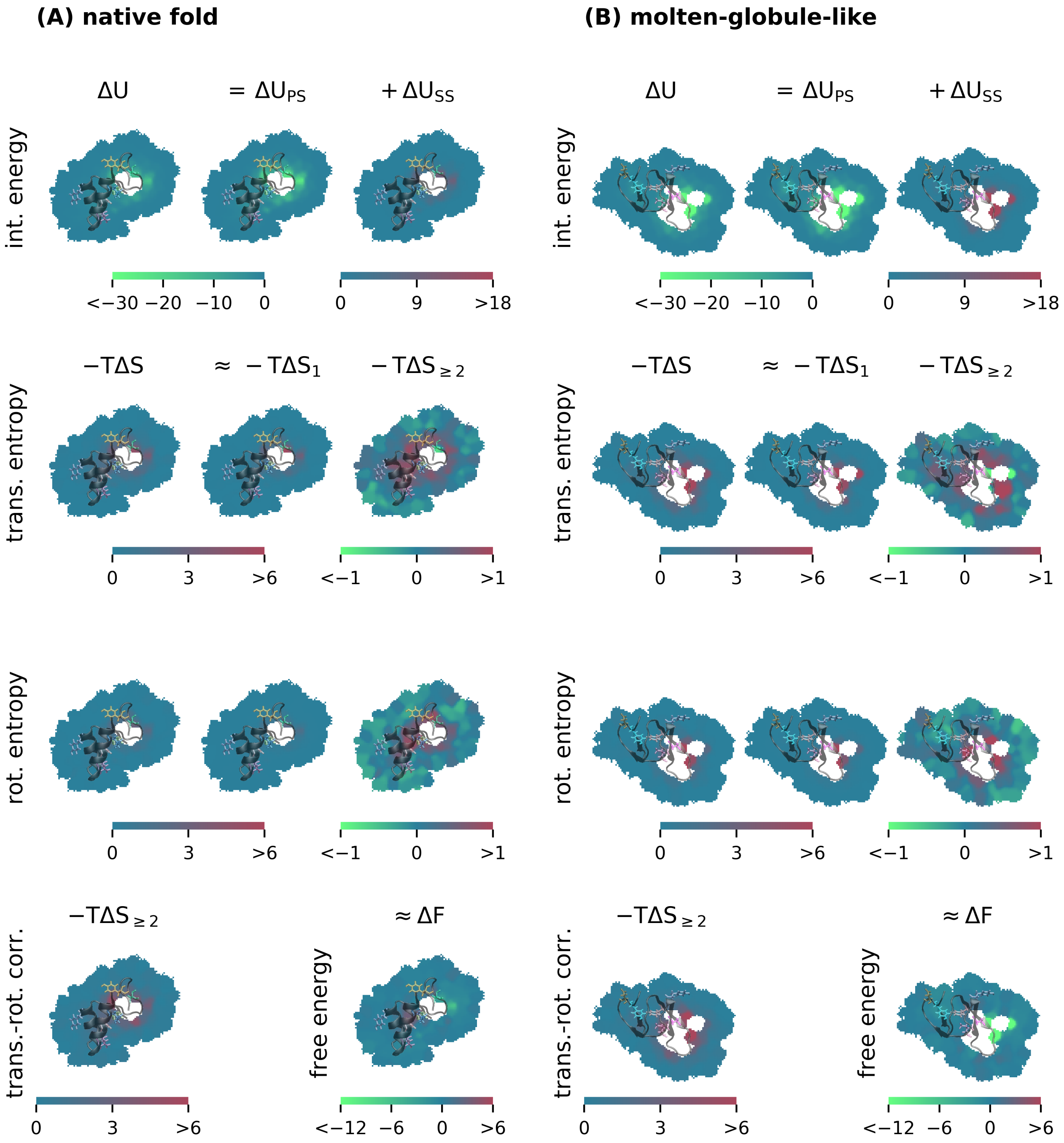

Figure S7: Spatially resolved solvent enthalpies, entropies, and free energies of Crambin in the native fold (A) and a molten-globule-like conformation (B), as shown in Fig. 2 of the main text. Visualization-slice across the three-dimensional space offset by ≈ -1.0 nm relative to the center of the protein.

REFERENCES

1. Dias, C. L., T. Ala-Nissila, J. Wong-ekkabut, I. Vattulainen, M. Grant, and M. Karttunen, 2010. The hydrophobic effect and its role in cold denaturation. *Cryobiology* 60:91–99.

2. Robertson, A. D., and K. P. Murphy, 1997. Protein structure and the energetics of protein stability. *Chemical reviews* 97:1251–1268.
