# Supplementary material for: Spatially resolved free-energy contributions of native fold and molten-globule-like Crambin
Source: Biophys J. 2021 Jun 2;120(16):3470–82. doi: 10.1016/j.bpj.2021.05.019 (PMC8391029; doi:10.1016/j.bpj.2021.05.019)
Supplement: Document S2. Article plus supporting material [file mmc2.pdf]

Biophysical Journal
Article

Biophysical Society

Spatially resolved free-energy contributions of native fold and molten-globule-like Crambin

Leonard P. Heinz[1,*] and Helmut Grubmüller[1,*]
[1]Department of Theoretical and Computational Biophysics, Max Planck Institute for Biophysical Chemistry, Göttingen, Germany

ABSTRACT The folding stability of a protein is governed by the free-energy difference between its folded and unfolded states, which results from a delicate balance of much larger but almost compensating enthalpic and entropic contributions. The balance can therefore easily be shifted by an external disturbance, such as a mutation of a single amino acid or a change of temperature, in which case the protein unfolds. Effects such as cold denaturation, in which a protein unfolds because of cooling, provide evidence that proteins are strongly stabilized by the solvent entropy contribution to the free-energy balance. However, the molecular mechanisms behind this solvent-driven stability, their quantitative contribution in relation to other free-energy contributions, and how the involved solvent thermodynamics is affected by individual amino acids are largely unclear. Therefore, we addressed these questions using atomistic molecular dynamics simulations of the small protein Crambin in its native fold and a molten-globule-like conformation, which here served as a model for the unfolded state. The free-energy difference between these conformations was decomposed into enthalpic and entropic contributions from the protein and spatially resolved solvent contributions using the nonparametric method Per|Mut. From the spatial resolution, we quantified the local effects on the solvent free-energy difference at each amino acid and identified dependencies of the local enthalpy and entropy on the protein curvature. We identified a strong stabilization of the native fold by almost 500 kJ mol^{-1} due to the solvent entropy, revealing it as an essential contribution to the total free-energy difference of (53 $\pm$ 84) kJ mol^{-1}. Remarkably, more than half of the solvent entropy contribution arose from induced water correlations.

SIGNIFICANCE The free-energy difference between folded and unfolded states governs the thermodynamic stability of a protein. Effects such as cold denaturation provide evidence that solvent-related contributions to the free-energy difference strongly stabilize the native protein fold. Quantifying the solvent contribution and its dependency on the individual amino acids is therefore essential for a better understanding of the protein folding thermodynamics. The obtained spatial resolution of solvent free-energy contributions might furthermore be relevant for protein design.

INTRODUCTION

Folding free energies of proteins at room temperature typically range in the order of a few tens of kilojoules per mol (1–4), which approximately corresponds to the interaction energy of just a few hydrogen bonds (5). This small folding free energy results from a delicate balance between competing enthalpy and entropy contributions, each of which are much larger in magnitude but compensate each other almost entirely (2). Shifting the balance by external factors, e.g., because of a mutation of an amino acid or simply by changing the temperature, can lead to drastic and sometimes counterintuitive consequences. For example, proteins can unfold at low temperatures (2,6,7), although the protein-internal interaction energies favor the folded state and the entropic protein free-energy contribution $-T\Delta S$, which favors the unfolded state, should decrease in magnitude. This effect, known as cold denaturation, seems paradoxical only if the protein, but not the solvent, contributions are considered for the free-energy difference estimation. Indeed, cold denaturation has been attributed to a temperature-induced weakening of the hydrophobic effect (2), which arises from the thermodynamics of the solvation shell. The hydrophobic effect is known to be a major driving force for protein folding and stability (2,8–10).

Cold denaturation exemplarily illustrates the importance of solvation thermodynamics for protein stability. However, its quantitative role in relation to other free-energy

Submitted October 27, 2020, and accepted for publication May 21, 2021.
*Correspondence: lheinz@gwdg.de or hgrubmu@gwdg.de
Editor: Tamar Schlick.
https://doi.org/10.1016/j.bpj.2021.05.019
© 2021 Biophysical Society.
This is an open access article under the CC BY license (http://creativecommons.org/licenses/by/4.0/).

contributions, the molecular mechanisms of solvent-driven protein stability, and how the solvent thermodynamics is affected by individual amino acids are still largely unresolved. To study the effect of solvation on protein stability, a decomposition of the individual solvent- and protein-related free-energy contributions of a protein native fold and the unfolded state is required. To furthermore characterize the effect of individual amino acids on the solvent free-energy contribution, a spatially resolved map of local solvent enthalpies and entropies is needed.

Here, we address these questions using all-atom molecular dynamics (MD) simulations of the quite typical soluble protein Crambin, for which high-resolution structural data are available. Its small size furthermore allowed us to study the full hydration shell. Because a representative ensemble of the extremely broad distribution of unfolded protein configurations is not sufficiently sampled within the timescales of MD simulations, we assessed the free-energy difference between the native fold and a transiently stable molten-globule-like conformation. Although in a molten-globule-like state, hydrophobic collapse has already partially occurred, we still expect a solvent-induced thermodynamic driving force toward the native fold.

We decomposed the free-energy difference between the two conformations into enthalpy contributions from protein-protein, protein-solvent, and solvent-solvent interactions and entropy contributions from both solvent and protein. To accurately compare the spatially resolved solvent entropy contributions to their respective enthalpic terms, we preferred a nonparametric method that also captures the entropy effects of multibody correlations. We therefore selected Per|Mut (11–14) to calculate the spatially resolved solvent entropy over alternative methods, such as the grid inhomogeneous solvation theory (15–21), which usually excludes multibody correlations, and the grid cell theory (22) or three-dimensional two-phase thermodynamics (23–25), which both rely on strong model assumptions. Per|Mut provides a further solvent entropy decomposition into the entropy of the individual water molecules and the entropy changes arising from multibody water-water correlations from both translational and rotational degrees of freedom. From the decomposition, we identified a substantial stabilizing effect of the solvent free-energy contributions of almost 500 kJ mol^{-1}. The further breakdown into different interaction energies and entropic water correlation terms allowed for an interpretation of the free-energy changes on a molecular level. Here, a significant increase of water-water correlations around the molten-globule-like conformation compared to the native fold was found, which corresponds to a stabilizing entropic free-energy contribution. From the spatial resolution of both solvation enthalpy and entropy, we obtain the local free-energy changes due to specific amino acid side chains and capture free-energy effects of the protein shape.

MATERIALS AND METHODS

MD simulations

All MD simulations were carried out using the software package Gromacs 2018 (26–30), the CHARMM36m force field (31–33) with the TIP3P water model (34) (CHARMM version), and the leapfrog integrator with a 2 fs time step. Unless stated otherwise, the temperature was kept at 300 K using the V-rescale thermostat (35) with a time constant of 0.1 ps. For equilibration simulations under NPT (fixed number of particles, constant pressure, and constant temperature) conditions, the Berendsen barostat (36) at 1 bar pressure with a time constant of 0.5 ps was used. All production runs were carried out using the Parrinello-Rahman barostat (37,38) with a time constant of 1.0 ps and 1 bar pressure. During all simulations, a short-range Coulomb cutoff of 1.2 nm was used; long-ranged electrostatic interactions were calculated using the particle-mesh Ewald method (39). Lennard-Jones forces (40) were switched smoothly between 1.0 and 1.2 nm. Bond-constraints were imposed on all water molecules using the SETTLE algorithm (41) and on all other bonds to hydrogen atoms using the LINCS algorithm (42). During production runs, configurations were stored every 10 ps for subsequent analysis.

System setup

A high-resolution structure of the protein Crambin (46 amino acids) was retrieved from the Protein Data Bank (1CBN) (43). The molecule was placed inside a cubic simulation box of size (7 nm)^3 and solvated with 10,950 water molecules. The system was then subjected to gradient descent energy minimization, which was terminated once the largest force on any atom decreased below 100 kJ mol^{-1} nm^{-1}. To equilibrate the solvent water, a 1 ns MD simulation under NVT (fixed number of particles, constant volume, and constant temperature) conditions was started, during which all heavy atoms of the protein were restrained using a force constant of 1000 kJ mol^{-1} nm^{-2}. To relax the box size to 1 bar, the system was subsequently simulated for 10 ns under NPT conditions, during which the restraints on the protein were maintained. Subsequently, the protein was equilibrated during a further 10 ns simulation under NVT conditions.

To obtain a molten-globule-like conformation of an unfolded state, the equilibrated protein was unfolded during 50 ns of heating and subsequent annealing. Here, the temperature was linearly increased to 600 K during the first 5 ns; the temperature was then kept at 600 K for the following 40 ns and eventually decreased back to 300 K during the last 5 ns. The unfolded structure was simulated for 2 μs under NPT conditions (Parrinello-Rahman barostat), during which a partial refolding occurred, resulting in the formation of two antiparallel β-sheets, shown in Fig. 1 *B*,

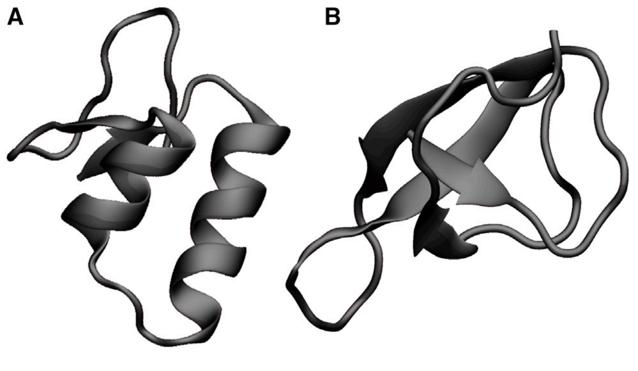

FIGURE 1 Ribbon-style visualization of Crambin in its native fold (*A*) and in a molten-globule-like conformation (*B*). Images were rendered using VMD (44). To see this figure in color, go online.

whereas the rest of the molten-globule-like conformation remained unstructured. To ensure a proper equilibration of the folded system, it was also simulated for further 2 μs.

Ensembles of four Crambin structures were retrieved for both the native fold and the molten-globule-like conformation by extracting equidistant frames from the last 3 ns of each of the equilibration runs. The ensembles therefore cover short-timescale processes such as the side chain reorientation but do not capture larger configurational motions, particularly of the molten-globule-like conformation.

A total of 10^5 statistically independent microstates, which are necessary to converge the nine-dimensional configurational subspace density estimates of the three-body correlations, were obtained at 10 ps intervals from 1 μs production runs under NPT conditions for each of the eight replicas. To restrict configurational changes of the protein, each atom was restrained using a force constant of 1000 kJ mol^{-1} nm^{-2}.

Entropy calculation

Hydration entropies were calculated from the production runs of the eight replicas using the method Per|Mut (13,14). For each replica, a permutation reduction (11,12) was carried out, which enhances sampling of the high-dimensional water configuration space by the Gibbs factor $N!$ and localizes the water molecules without changing their physical and statistical properties. The first trajectory frame was used as the reference configuration.

Spatially resolved entropies were calculated for the 4000 water molecules closest to the protein using a third-order mutual information (MI) expansion (45–48). Pairwise MI terms of the translational and rotational entropy, as well as for the translation-rotation correlation, were calculated for water molecules with a maximal average distance of 1.0 nm. For third-order terms, a 0.33 nm cutoff was used. The individual entropy terms in the expansion were calculated using a k-nearest-neighbor algorithm with a value of $k = 1$ for all expansion orders. The entropy of the solvation shell consisting of the closest 1000 molecules was used for analysis. From those results, the entropy of the inner solvation shell, consisting of the closest 1000 molecules (equivalent to a thickness of ~0.8 nm), was calculated as $S_{\text{internal}} - I/2$. Here, S_{internal} is the sum of all expansion terms concerning molecules from the inner solvation shell and I is the sum of all MI terms with a molecule inside and a molecule outside the inner solvation shell. Error bars were obtained as standard deviations of the four replicas per state.

Protein entropies were estimated for both states using Schlitter's method (49) on all nonhydrogen protein atoms from additional unrestrained MD simulations. Here, the molten-globule-like conformation partially refolded after 1.5 μs (see Fig. S1). To obtain values consistent with the restrained states, for which the remaining free-energy terms were calculated, the analysis was limited to the first 1 μs of the trajectories. Errors were estimated from a second analysis using only 0.5 μs of the trajectories.

Enthalpy calculation

Enthalpies were calculated as $U + pV$, where U is the sum of all interaction energies, averaged over the MD trajectory. The work term pV was included as the product of pressure (1 bar) and average box volume.

For each water molecule of the inner solvation shell, the interaction energies with the protein, the outer solvation shell, and all other molecules of the inner shell were calculated. Lennard-Jones parameters and partial charges were taken from the CHARMM36m force field, and a 2 nm cutoff was used for Lennard-Jones interactions. The interaction energy of the protein was calculated directly with the software package Gromacs.

Enthalpy and interaction energy differences were then calculated with respect to bulk water (Figs. 2 and 3), as well as between the native fold and molten-globule-like configurations (Fig. 4). Interaction energy differences were defined as the difference between the mean interaction energies in each of two considered states.

Visualization of entropy and enthalpy

The entropy and interaction energy contributions of each molecule were calculated by splitting the two-body MI terms, three-body MI terms, and the pairwise interaction energies equally between the respective molecules, accounting for the otherwise inherent double or triple-counting (50) of pairwise interaction energies as well as two- and three-body correlations. The simulation box was then divided by a 129 × 129 × 129 grid. The entropy and energy values of each voxel were calculated as the average contribution of all water molecules that visited the voxel, weighted by the number of times the voxel was visited by each water molecule. Because of permutation reduction, each voxel was only visited by a few of the localized water molecules.

Error estimation

Errors of solvent entropy and solvent enthalpy contributions of each water molecule were estimated as standard deviation of bulk-phase water molecules, assuming that water molecules close to the solvent are subject to the same spread (14). The estimated errors are listed in Table 1.

Here, U_{SS} is the solvent-solvent interaction energy, and $-TS^{\text{trans}}$ and $-TS^{\text{rot}}$ are the translational and rotational free-energy contributions, respectively. Both are split into a single-body term $-TS_1$ and a multibody term $-TS^{\geq 2}$. $-TS_{\geq 2}^{\text{trans-rot}}$ corresponds to the entropy contribution from translation-rotation correlations.

Residue contributions and convexity

The local interaction energy and the local solvation entropy contribution around each amino acid were calculated as the average contribution of all water molecules within 0.4 nm of the amino acid. Here, the distance was measured as the minimal distance to any atom of the residue. The contribution of the pV term was small compared to the other contributions (19.8 kJ mol^{-1} for both the native fold and molten-globule-like conformations) and was therefore neglected. The local free energy was thus calculated as the sum of the interaction energy and the entropy contribution.

As a measure of the local topology, the convexity of the protein-solvent surface was calculated for each residue as

$$c = \frac{N_d}{\left(\frac{4}{3}\pi d^3 - V_{\text{res}}\right) n_W},$$

where N_d is the number of water molecules within a radius of $d = 1$ nm around the amino acid, V_{res} is the volume of the residue itself, and n_W is the number density of water.

Accordingly, a convexity of $c = 0$ indicates that the entire volume within the radius d around the residue is occupied by other protein atoms, i.e., that the residue is buried. Conversely, a value of $c = 1$ shows that the residue is fully exposed to the solvent.

To quantify the hydrophobicity of a residue and for comparison with our calculations, the empirical hydrophobicity scale by Zhao et al. (51) was used; a small value indicates a hydrophilic amino acid, whereas a large value denotes hydrophobicity.

RESULTS AND DISCUSSION

Spatially resolved solvation free-energy contributions

To quantify and characterize the solvent contribution to the stability of the protein Crambin, we carried out and

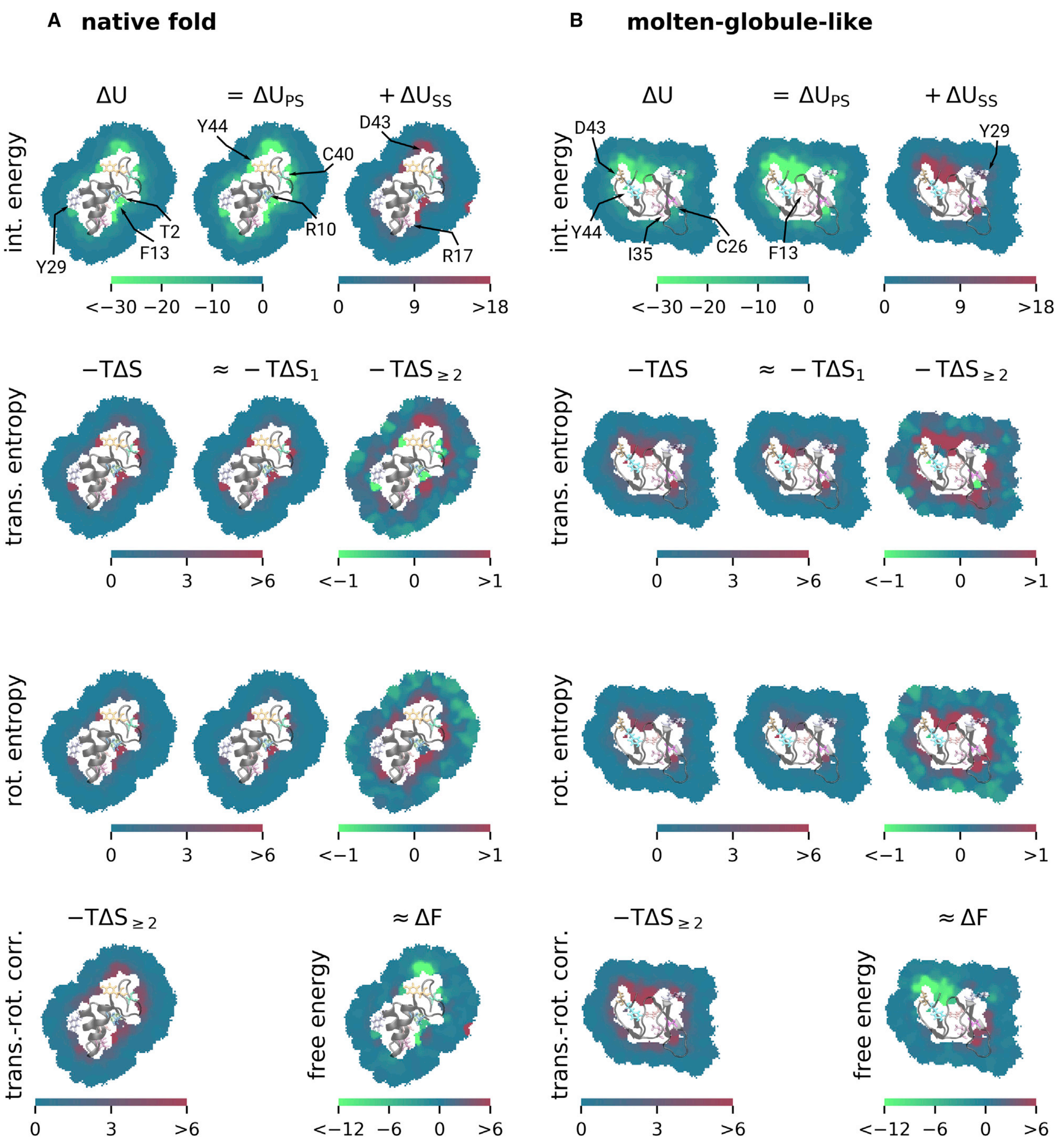

FIGURE 2 Spatially resolved hydration free-energy decomposition of Crambin in its native fold (*A*) and the molten-globule-like conformation (*B*) relative to bulk water, visualized on a representative two-dimensional slice through the center of the molecules. The total interaction energy ΔU (*top row*, *left*) is split into protein-solvent interactions ΔU_{PS} (*top row*, *center*), and solvent-solvent interactions ΔU_{SS} (*top row*, *right*). In a similar manner, the translational and rotational entropy contributions $-T\Delta S$ (*center rows*) are split into the single-molecule entropy $-T\Delta S_1$ and multibody correlations $-T\Delta S_{\geq 2}$. The entropy contribution from the translation-rotation correlation is shown in the bottom left. The spatially resolved free-energy change (sum of the *first column*) is shown in the bottom right. All numerical values are given in kJ mol^{-1}, and important residues are marked by arrows. To see this figure in color, go online.

compared MD simulations of the native fold and a molten-globule-like protein conformation, as described in the Materials and methods. We will first address the contribution of individual amino acids and the protein shape to the solvent free-energy contributions for a number of exemplary residues and subsequently provide a more systematic assessment. To this end, we calculated spatially resolved solvent interaction energies and corresponding entropies for both the native fold and the molten-globule-like conformation, respectively.

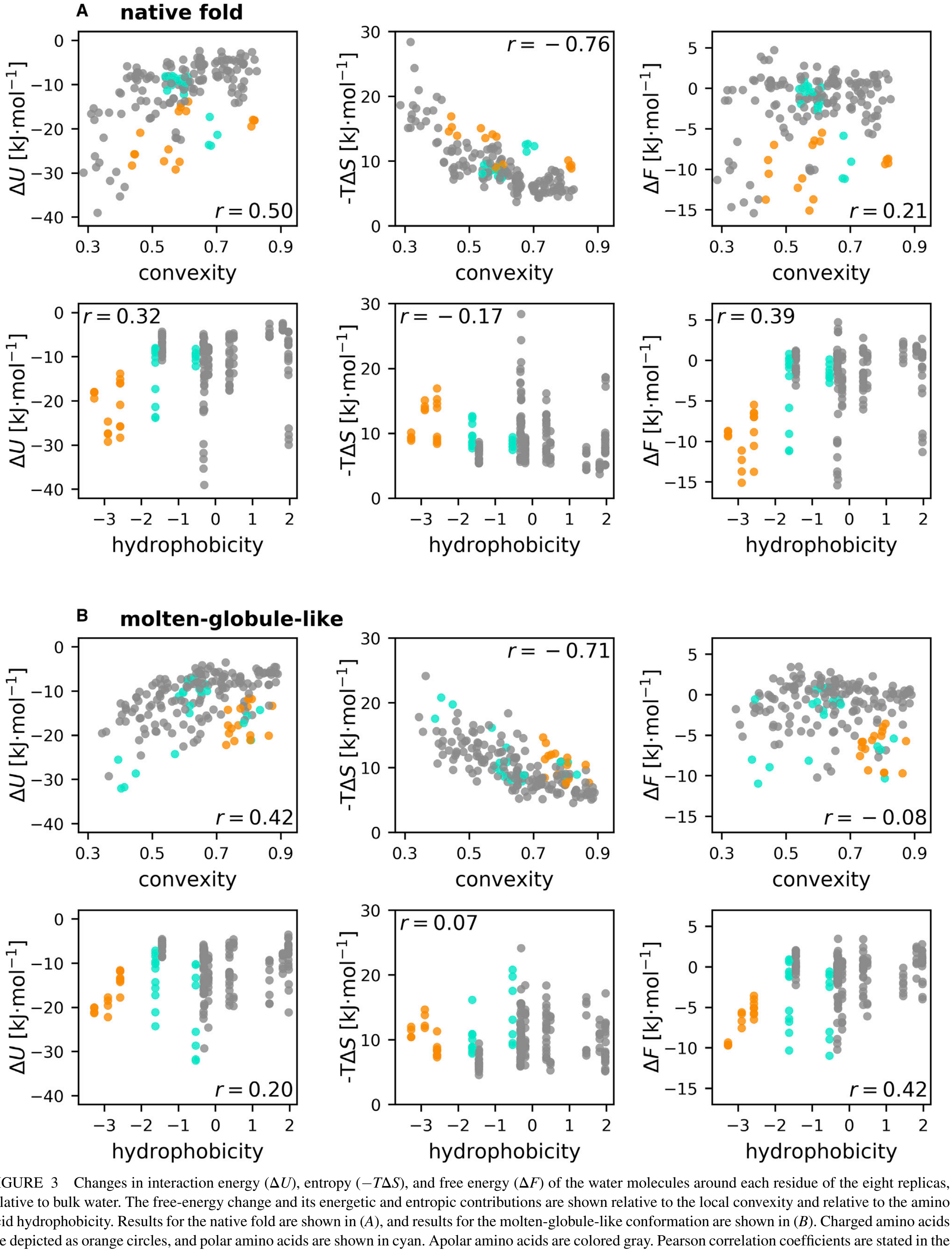

FIGURE 3 Changes in interaction energy (ΔU), entropy ($-T\Delta S$), and free energy (ΔF) of the water molecules around each residue of the eight replicas, relative to bulk water. The free-energy change and its energetic and entropic contributions are shown relative to the local convexity and relative to the amino acid hydrophobicity. Results for the native fold are shown in (*A*), and results for the molten-globule-like conformation are shown in (*B*). Charged amino acids are depicted as orange circles, and polar amino acids are shown in cyan. Apolar amino acids are colored gray. Pearson correlation coefficients are stated in the corners of each plot. To see this figure in color, go online.

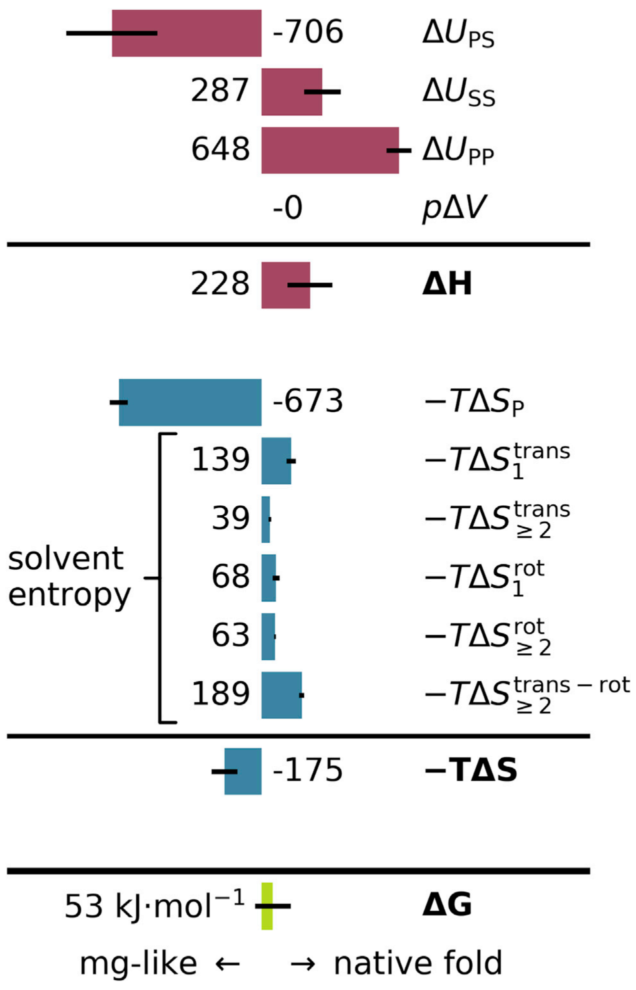

FIGURE 4 Decomposition of the folding free energy (*green*) into enthalpy (*red*) and entropy terms (*blue*). Positive values favor the native fold, negative values favor the molten-globule-like conformation. Values in kJ mol^{-1}. Error bars denote errors of the mean of the 2 × 4 replicas. To see this figure in color, go online.

Because any spatially resolved picture of the solvation shell thermodynamics depends on and changes with the particular conformation of the protein, we chose a representative conformation for each of the folded and the molten-globule-like ensembles. To obtain better statistics, we carried out simulations of four similar replicas for each conformation. Because the molten-globule-like conformation is structurally unstable, we restrained the structures as described in the Materials and methods.

For each of the 2 × 4 replicas, spatially resolved solvent interaction energies ΔU were calculated directly from the average interaction energies between protein and solvent (ΔU_{PS}) and between the solvent molecules (ΔU_{SS}), assuming pairwise interaction energy functions. Solvent entropy contributions were calculated using the method Per|Mut (13,14), which yields a spatially resolved entropy decomposition into terms S_1^{trans}, S_1^{rot}, $S_{\geq 2}^{trans}$, $S_{\geq 2}^{rot}$, and $S_{\geq 2}^{trans-rot}$. Here, S_1^{trans} and S_1^{rot} are the translational and rotational entropies of the individual water molecules, ignoring any correlations. $S_{\geq 2}^{trans}$ and $S_{\geq 2}^{rot}$ capture the entropy loss due to two- and three-body correlations between water molecules for translational and rotational degrees of freedom, respectively. Higher-order correlations were assumed to be negligible to approximate the solvent entropy. A positive value of $-T\Delta S_{\geq 2}$ denotes an unfavorable free-energy increase relative to bulk water because of increased water-water correlations. Conversely, a negative value of $-T\Delta S_{\geq 2}$ indicates a favorable free-energy contribution because of weakened correlations relative to bulk water. Similarly, $\Delta S_{\geq 2}^{trans-rot}$ is the entropy change due to correlations between translational and rotational degrees of freedom of water molecules. Note that contrary to other decompositions of entropy (52–54), $-T\Delta S_1$ and $-T\Delta S_{\geq 2}$ are not the associated entropy differences to changes of the protein-solvent (U_{PS}) or solvent-solvent (U_{SS}) interaction energies, respectively, but provide an independent decomposition into single-body entropies and multibody correlations.

TABLE 1 Estimated solvent enthalpy and solvent entropy errors per water molecule

Contribution	Error [kJ mol^{-1}]
U_{SS}	1.05
$-TS^{trans}$	0.46
$-TS_1^{trans}$	0.09
$-TS_{\geq 2}^{trans}$	0.37
$-TS^{rot}$	0.22
$-TS_1^{rot}$	0.02
$-TS_{\geq 2}^{rot}$	0.22
$-TS_{\geq 2}^{trans-rot}$	0.18

Fig. 2 compares these spatially resolved free-energy contributions for the native fold (Fig. 2 *A*) and the molten-globule-type conformation (Fig. 2 *B*), respectively. As shown in the upper left of Fig. 2 *A*, the interaction energy ΔU of water molecules near the protein is generally more negative (stronger) than in bulk water, particularly close to charged residues such as R17 and D43, with interaction energy differences of −48 and −27 kJ mol^{-1}, respectively. Similarly, in the vicinity of the polar hydroxyl groups of the tyrosine residues Y29 and Y44, the energy is lower than in bulk water (ΔU = −22 kJ mol^{-1}), and close to residue C40, the interaction energy difference is −27 kJ mol^{-1}. Residues T2, F13, and R10 form a "groove" in the native fold structure, with strong interaction energies below −30 kJ mol^{-1} relative to bulk water.

Fig. 2 *A* (*top row*, *center* and *right*) reveals the individual interaction energy contributions from the interactions between protein and solvent ΔU_{PS}, as well as from interactions between solvent molecules ΔU_{SS}. The interaction energy of hydration is dominated by the protein-solvent interactions, in which charged and polar residues show particularly favorable interactions. Although solvent-solvent interactions (*top row*, *right*) have no net effect on the overall free-energy difference (52–54), they partially counteract the locally

favorable protein-solvent contributions and therefore contribute to the total enthalpy component. Here, water molecules that bind to charged residues perturb the hydrogen bond network of the surrounding water, thereby weakening the solvent-solvent interactions. Accordingly, around the charged residues R10, R17, and D43, the solvent-solvent interaction energy becomes strongly unfavorable with values between +17 and +30 kJ mol^{-1} relative to bulk water. Similarly, but to a lesser extent, solvent-solvent interactions are weakened for water molecules around the hydroxyl groups of residues Y29 and Y44, resulting in unfavorable energy changes ranging between +10 and +15 kJ mol^{-1}.

Next, we quantified the impact of these strong interactions on the local entropy. For the charged residues R10, R17, and D43, as well as for the polar residues Y29 and Y44, both the translational and rotational solvent entropies are reduced (Fig. 2 *A*, *center rows*). At these sites, the free-energy contributions $-T\Delta S$ vary from +5.5 to +12 kJ mol^{-1} (translational) and from +3.5 to +8.2 kJ mol^{-1} (rotational) and therefore weaken the otherwise strong solvation. The entropy loss is dominated by the first-order terms ΔS_1^{trans} and ΔS_1^{rot}, revealing that the reduced mobilities of the individual water molecules due to the protein (partial) charges, and not water-water correlations, are the main cause of the entropy loss.

Translational entropy free-energy contributions from multibody correlations $-T\Delta S_{\geq 2}$ very close to the charged and polar protein parts are indeed relatively weak, but not negligibly so, with values between −2.0 and −1.5 kJ mol^{-1} relative to bulk water. We note that the water-water correlations, here quantified by $-T\Delta S_{\geq 2}$, differ from the solvent-solvent entropy contribution, as defined, e.g., by Ben-Naim (52,53) and Yu et al. (54), and hence, our results are compatible with their finding that the latter is exactly compensated by the respective enthalpy term. Remarkably, for second-shell water molecules around charged residues, these correlations again change sign, e.g., for residues R10 (+1.0 kJ mol^{-1}), D43 (+1.2 kJ mol^{-1}), and Y44 (+0.6 kJ mol^{-1}), thus being less favorable compared to bulk water. A similar effect is seen for the rotational multibody correlations in which the free-energy contribution almost vanishes at the first hydration shell (e.g., +0.2 kJ mol^{-1} at R10 and +0.4 kJ mol^{-1} at Y29 and Y44, each with an estimated error of 0.22 kJ mol^{-1}) but increases to values from +0.7 to +1.0 kJ mol^{-1} within the second hydration shell.

A possible explanation for this effect is that the strong binding of the first-shell water molecules (for rotational degrees of freedom, the strong directionality due to the water dipole moment (55)) results in reduced fluctuations, which limits the correlations with the remaining water molecules (for a more detailed analysis, see Supporting materials and methods). As a result, the multibody correlation entropy contribution is more favorable for the bound molecules compared to bulk water. In contrast, the fluctuations of the second-shell water molecules are larger because of the reduced electrostatic interactions with the charged and polar residues, as shown by bulk-like single-body entropy terms. Therefore, the correlations with the adjacent water molecules reduce the entropy by a larger amount.

As the last remaining entropy term, we also quantified the entropy reduction due to translation-rotation correlations (Fig. 2 *A*, *lower left*), which reveals increased correlations close to the protein surface, particularly in the vicinity of charged residues and polar chemical groups. Here, the respective solvent free-energy contribution is increased by +3.2 kJ mol^{-1} (R10), +5.9 kJ mol^{-1} (R17), +2.7 kJ mol^{-1} (Y29), +5.0 kJ mol^{-1} (D43), and +4.0 kJ mol^{-1} (Y44) compared to bulk water. The correlations and the corresponding unfavorable free-energy changes arise because the strong directionality of the water molecules close to a protein (partial) charge quickly decreases with distance. A water molecule close to a charged amino acid is therefore, on average, more strongly oriented than a water molecule at a larger distance.

To characterize the local solvent free-energy change relative to bulk water, we considered the sum of all free-energy contributions (Fig. 2 *A*, *bottom right*). Close to hydrophilic residues, enthalpic contributions dominate, such that the total free-energy change is favorable, as expected. The water molecules that form hydrogen bonds to the hydroxyl groups of Y29 and Y44 contribute −5.3 and −6.2 kJ mol^{-1} to the hydration free-energy difference of the protein, respectively. Around the charged residues R17 and D43, the free energy per water molecule is reduced by −17.5 and −32 kJ mol^{-1}, respectively. Close to the residue C40, the free energy is reduced by −5.4 kJ mol^{-1}, and in the "groove" formed by residues T2, R10, and F13, the free-energy contribution per water molecule is −6.6 kJ mol^{-1} compared to bulk water. Here, the average interaction energy of the closest water molecule to the three amino acids is +4, −57, and 0 kJ mol^{-1}, respectively. Although the entropic contributions cannot be assigned to specific residues, their total contribution to the free energy (~30 kJ mol^{-1}) is smaller than the enthalpic contribution of R10. In this sense, the favorable free energy is enthalpically dominated because of the charged residue R10. Because of the water-water correlation effects discussed above, second-shell water molecules around protein charges contribute unfavorably to the hydration free energies, with values ranging from +1.8 to 3.2 kJ mol^{-1} per molecule.

Next, we carried out the same analysis for a molten-globule-like conformation to identify possible differences in the solvation thermodynamics between the two states. As shown in Fig. 2 *B*, the enthalpic and entropic changes for water molecules close to charged and polar residues, e.g., for Y29, D43, and Y44, are similar to the native fold. However, the spatial distribution of the individual free-energy contributions is different. Whereas in the folded state, prominent differences of the local free-energy contributions

are isolated and can be well attributed to individual (charged or polar) residues, a particularly large region of favorable interaction energies and unfavorable entropies is seen around residues D43, Y44, and F13 of the molten-globule-like fold. Here, we considered two possibilities: first, the (coincidental) colocalization of specific amino acids in the molten-globule-like state could give rise to the spread-out distribution. Alternatively, the locally concave shape of the molten-globule-like conformation could provide a possible explanation of the large volume of the affected region. In the latter case, the semicavity exposes a markedly larger surface area to the solvent, which strengthens the solvent-protein interactions (-50 kJ mol^{-1} per molecule) but weakens the solvent-solvent interactions ($+20$ kJ mol^{-1}) as the number of neighboring water molecules is reduced because of the protein geometry. Correspondingly, the concave shape restricts the mobility of the water molecules, resulting in an unfavorable entropy change compared to bulk water ($+5.8$ kJ mol^{-1} translational, $+5.0$ kJ mol^{-1} rotational). Indeed, similar entropy and enthalpy effects were also seen to a smaller extent at the concave "groove" formed by residues T2, R10, and F13 in the native fold. To probe whether this observation is anecdotal, we systematically compared the local curvature to the local free-energy contributions of all amino acids in the next subsection.

In addition to the single-body entropy reduction in the concavity, we also observed unfavorable entropy changes due to the multibody correlation terms, which contribute $+2.2$ kJ mol^{-1} (translational), $+2.1$ kJ mol^{-1} (rotational), and $+9.5$ kJ mol^{-1} (translation-rotation correlation). In light of the geometry-induced weaker solvent-solvent interactions, such high correlations are unexpected. To check whether this observation is anecdotal or, alternatively, more general, we systematically compared the multibody entropy terms to the local convexity for each amino acid of the 2×4 replicas. As shown in Fig. S2, there are indeed stronger correlations (i.e., unfavorable free-energy contributions) between rotational degrees of freedom ($-T\Delta S^{\text{rot}}_{\geq 2}$) and between translational and rotational degrees of freedom ($-T\Delta S^{\text{trans-rot}}_{\geq 2}$) for smaller convexity values (i.e., locally more concave surfaces). Translational correlations ($-T\Delta S^{\text{trans}}_{\geq 2}$) show an inverted dependence on the local convexity. So far, we are unable to provide an explanation for these effects.

For the native fold, as well as for the molten-globule-like conformation, the well-known tug of war between enthalpic and entropic free-energy contributions results in a partial compensation of the two contributions (56). For the native fold, this compensation often also applies to the multibody correlation entropies and the solvent-solvent interaction energies, e.g., close to residues Y29, Y44, and R10. However, this compensation of solvent-solvent terms is not seen for water molecules in the concavity of the molten-globule-like conformation, which might be an effect of the concave shape of the protein. Despite the partial enthalpy-entropy compensation, the solvent free energy is mainly affected by the protein-solvent interaction energies.

The entropy contributions in the native fold state mainly originate from water molecules that are bound to protein charges and mostly affect the first hydration shell. To the contrary, the entropy contributions in the molten-globule-like state are more spread out, and the second hydration shell is also significantly affected. This observation might be a direct consequence of the hydrophobic driving forces of protein folding (9), in which hydrophobic residues, which result in increased multibody correlation entropies (14,57,58), are predominantly packed into the protein interior of the native fold.

We furthermore note that the water entropy seems to become more unfavorable in concave parts of the molecules, for example, as seen in the "groove" at residues T2, R10, and F13 for the native fold and around residues R13, D43, and Y44 for the molten-globule-like conformation. The water interaction energy seems to show a compensating effect.

Residue contributions

So far, we have focused on a few illustrative example residues. For a more comprehensive and systematic assessment of the protein-shape effect on each of the local free-energy contributions, we compared the free energies close to all residues to the local convexity, calculated as described in Residue contributions and convexity. Here, the interaction energies and solvation entropies associated to each amino acid were calculated as the average contribution of all water molecules within 0.4 nm of the amino acid. The reported results therefore depend on this cutoff, which was chosen to correspond to the range within which water molecules were affected by amino acids, as shown in Fig. 2. To distinguish protein topology effects from those of the solvation properties of the individual amino acids, we furthermore compared the local free-energy changes around each residue to its hydrophobicity index (51).

Fig. 3 reveals a clear correlation between the local interaction energy differences with respect to bulk water and the convexity for both the native fold (Fig. 3 *A*) and the molten-globule-like states (Fig. 3 *B*), reflected in Pearson correlation coefficients of 0.50 and 0.42, respectively. Here, the interaction energy of the water molecules at a concave surface (convexity ≈ 0.3) is reduced by up to 40 kJ mol^{-1} for the native fold and by up to 33 kJ mol^{-1} for the molten-globule-like fold, whereas the interaction energy of water molecules at convex surfaces of the protein (convexity $>$ 0.8) only differs by a small amount ($\Delta U > -5$ kJ mol^{-1}) relative to bulk water. This effect is to be expected, as more solvent-exposed residues interact with a larger number of water molecules.

An even stronger but inverse correlation (correlation coefficients of -0.76 and -0.71 for the native fold and the molten-globule-like states, respectively) is seen in Fig. 3

for the solvation entropy contribution $-T\Delta S$; here, the water entropy becomes strongly unfavorable at concave surfaces, with contributions of up to 28 kJ mol^{-1} for the native fold and of 24 kJ mol^{-1} for the molten-globule-like configurations. Correspondingly, the entropy contributions at convex surfaces are small ($-T\Delta S < 5$ kJ mol^{-1}). This observation is in line with previous reports about unfavorable water entropies and favorable water enthalpies in cavities (59,60) and altered water behavior at concave surfaces (61).

Interestingly, the convexity dependencies of enthalpy and entropy almost compensate each other, such that the local free energy, shown on the top right of Fig. 3, *A and B*, shows no significant dependency on the surface convexity ($r =$ 0.21 and -0.08 for the native fold and the molten-globule-like state, respectively). Furthermore, the side chain polarity, as indicated by the color code in Fig. 3, has no significant impact on the convexity effects of interaction energy and entropy.

As shown in the bottom rows of Fig. 3, *A and B*, there is only a modest correlation of the local interaction energy and entropy contributions with the hydrophobicity index of each amino acid ($r = 0.32$ and 0.20 for native fold and molten-globule-like state, respectively). However, as expected, the local free energy correlates more strongly with the hydrophobicity index ($r = 0.39$ and 0.42 for native fold and molten-globule-like state, respectively), in which the most hydrophilic residues show favorable local solvation free energies of -16 to -5 kJ mol^{-1}, whereas local free-energy changes of -11 to $+3$ kJ mol^{-1} are attributed to hydrophobic residues. Unexpectedly, for the nonpolar residues, no correlation between their solvation free energies in the protein context and their hydrophobicity index is seen. As shown in the bottom right of Fig. 3, *A and B*, the most favorable free energy is observed around charged residues (colored *orange*), with a free-energy change of ($-8.1 \pm$ 2.3) kJ mol^{-1}. Around polar residues (colored *cyan*), the average free-energy change is (-3.14 ± 3.72) kJ mol^{-1}; around apolar residues (colored *gray*), an average contribution of (-1.53 ± 4.45) kJ mol^{-1} is seen.

Comparing the native fold with the molten-globule-like conformation, the most striking difference is seen for the charged residues, for which the local free-energy contribution is more favorable for the native fold ((-9.64 ± 2.58) kJ mol^{-1}) compared to the molten-globule-like conformation ((-6.53 ± 1.97) kJ mol^{-1}). This finding agrees with the well-known and important mechanism of stabilizing a folded state by an optimized geometry that maximally exposes hydrophilic charged residues.

Free-energy decomposition

To obtain a quantitative understanding of the overall hydration contribution to protein stability, we decomposed the total free-energy difference $\Delta G = G_{\mathrm{m}} - G_{\mathrm{f}}$ (following Dias et al. (2)) of the molten-globule-like conformation and the native fold into individual free-energy contributions. In addition to solvent contributions, we also calculated protein interaction energies U_{PP} directly from the ensemble of the eight restrained replicas, as well as protein entropies S_{P} from unrestrained MD simulations of the native fold and the molten-globule-like conformation, respectively. To ensure that the unrestrained simulation of the molten-globule-type only system samples the conformation of the four similar molten-globule-like ensemble members used for the calculation of the other free-energy contributions, the simulation time was restricted to 1 μs. During this time, the conformation did not change markedly, with a root-mean-square deviation below 0.7 nm. Accordingly, our free-energy budget includes only part of the conformational entropy of the whole molten-globule-type ensemble.

Fig. 4 summarizes the calculated differences between the native fold and the molten-globule-like conformation for the various free-energy contributions. Positive values indicate a stabilization of the native fold, and negative values favor the molten-globule-like conformation. Here, absolute free energies, enthalpies, and entropy contributions were calculated for each of the 2×4 replicas separately, from which average differences between the two states were then obtained. As expected, we obtained a total free-energy difference that favors the folded state ((53 $\pm$ 84) kJ mol^{-1}). Although, to the best of our knowledge, a measured value is not available, this result agrees with the expected range of a few tens of kilojoules per mol (2,3).

Likely because the molten-globule-like conformation has a larger solvent-accessible surface area ((36.9 $\pm$ 0.6) nm^2 vs. (31.3 $\pm$ 0.3) nm^2 for the native fold), the total interaction energy between the protein and inner solvent shell U_{PS} destabilizes the native fold by ($-706 \pm$ 216) kJ mol^{-1} with respect to the molten-globule-like conformation. In contrast, the interaction energies within the inner solvation shell (closest 1000 molecules), given by $\Delta U_{\mathrm{SS}} = (287 \pm 86)$ kJ mol^{-1}, partially compensate the destabilizing contribution of ΔU_{PS}. The work term $p\Delta V = (-0.002 \pm 0.002)$ kJ mol^{-1} shows no significant difference between the two conformations. As expected, the internal interaction energies of the protein strongly stabilize the folded state ($\Delta U_{\mathrm{PP}} = (648 \pm 59)$ kJ mol^{-1}). Overall, the enthalpy contributions strongly favor the folded state ($\Delta H = (228 \pm 106)$ kJ mol^{-1}), in line with textbook thermodynamics of protein folding.

Next, we quantified all entropic contributions to the free-energy difference, which are expected to largely compensate the strongly stabilizing enthalpy difference. Indeed, the protein entropy strongly favors the molten-globule-like state even though only one single metastable conformation was considered ($-T\Delta S_{\mathrm{P}} = (-673 \pm 42)$ kJ mol^{-1}). Crucially, however, all solvation entropy terms stabilize the native fold, adding up to 498 kJ mol^{-1}. One of the largest contributions is the singe-particle translational entropy $S_1^{\mathrm{trans}} = (139 \pm 22)$ kJ mol^{-1}. In contrast, the

respective rotational entropy contribution, which many studies focus on, yields only a difference of (68 ± 17) kJ mol^{-1}. Also intriguingly, the second- and third-order MI terms $S^{\mathrm{trans}}_{\geq 2}$ and $S^{\mathrm{rot}}_{\geq 2}$, which denote the two- and three-body correlations, contribute large differences of (39 ± 6) kJ mol^{-1} and (63 ± 4) kJ mol^{-1}, respectively. Strikingly, the largest solvent entropy contributions stem from the translation-rotation correlation entropy, which favors the native fold by (189 ± 12) kJ mol^{-1}. Obviously, these entropies, which arise from the correlated motion of water molecules, contribute markedly to the folding thermodynamics of Crambin.

Interestingly, partial error compensation between the free-energy terms shown in Fig. 4 results in a total free-energy error (±84 kJ mol^{-1}) that is smaller than the error of some of its components (e.g., ΔU_{PS} with an error of ±216 kJ mol^{-1}). We think that this is a result of the fact that free-energy differences typically converge faster than their individual components; however, this could also be a coincidental result of the limited sample size of four replicas for each of the two states.

In summary, the largest contribution to solvent-induced entropic stabilization (498 kJ mol^{-1}) of the native fold is not due to the mobility of single water molecules but to their correlated motion (291 kJ mol^{-1}). Conversely, the protein-solvent interaction energies ΔU_{PS} and the protein entropy ΔS_{P} are the only destabilizing terms.

CONCLUSIONS

To quantitatively assess the role of the protein structure and individual side chains in the solvation thermodynamics and stability of the native fold, we compared spatially resolved enthalpic and entropic solvation free-energy contributions of the native fold and the molten-globule-like state of the small globular protein Crambin. A systematic analysis of the solvation thermodynamics of all residues revealed that their local enthalpies and entropies are mainly affected by the protein charges and the local curvature of the protein surface. Close to charged or polar residues, a more favorable interaction energy and a less favorable entropy contribution were observed. Interaction energies are here dominated by protein-solvent terms, whereas solvent-solvent interactions are weakened and therefore become less favorable around protein charges.

Single-body entropy terms are strongly reduced close to protein charges, as the water molecules bind to the protein and therefore lose translational and orientational mobility. For solvent multibody correlation contributions, a more complex picture emerged. Whereas correlations with the remaining water molecules are weakened for the bound water molecules, the second hydration shell water molecules show an increased correlation and therefore an unfavorable multibody entropy contribution. The translation-rotation correlation entropy becomes unfavorable close to the protein surface, particularly close to protein charges, indicating that the correlation between translational and rotational degrees of freedom increases close to the protein surface.

The local contributions from enthalpy and entropy largely compensate each other, as expected, so that the local free-energy values are smaller in magnitude. The spatial distribution of the local free-energy changes is mainly dominated by favorable contributions close to polar or charged residues. This result suggests that, at least for Crambin, the solvation of hydrophilic residues contributes to a larger extent to the stability of the folded protein than burying hydrophobic residues into the protein interior. We furthermore observed that concave protein surfaces seem to cause more favorable hydration enthalpies and less favorable entropies.

To distinguish between a possible curvature effect and the influence of individual amino acids, we compared the local convexity of the protein surface and the hydrophobicity index of each protein residue to the local enthalpies, entropies, and free energies. Indeed, strong correlations between surface convexity and solvation interaction energies and entropies are seen. A more concave shape of the protein surface generally correlates with more favorable interaction energies and less favorable solvation entropy contributions. Because of the opposing effects on the two quantities, the local free energy of solvation remains almost unaffected by the protein curvature. As expected, we found more favorable local solvent free energies close to hydrophilic residues (i.e., those with a small hydrophobicity index) than close to hydrophobic residues. The finding indicates that the physicochemical properties of the respective amino acids have a larger effect on the local free energy than the protein curvature, although curvature strongly affects both entropy and enthalpy.

To see how much the solvation contributions weigh in the total free-energy balance, we decomposed the total folding free-energy difference between the native fold and molten-globule-like conformation of Crambin into individual enthalpy and entropy contributions from both protein and solvent. Although other computational methods, such as replica exchange MD (62–65), allow one to calculate ΔG with better precision and higher accuracy, our decomposition into individual energy and entropy terms provides additional insights into the thermodynamics of protein stability, even on the globular protein energy budget level. Specifically, the protein-solvent interaction energies and the protein entropy turn out to be the only destabilizing contributions to the free energy, whereas the solvent-related entropy contributions favor the folded state of Crambin by almost 500 kJ mol^{-1}. Furthermore, the solvent entropy difference is not dominated by water mobilities but by induced multibody water correlations.

One may ask whether this result is compatible with the well-known identity by Ben-Naim (52,53) and Yu et al. (54), who have shown that the solvent-solvent enthalpy change ΔH_{SS} (which here is approximately ΔU_{SS}, as

$p\Delta V \approx 0$) upon solvation compensates exactly the "solvent reorganization" entropy contribution (usually denoted ΔS_{SS}). In fact, our solvent correlation entropy contributions $-T\Delta S_{\geq 2}$ do not at all cancel with ΔU_{SS}, as one might expect. Note, however, that $-T\Delta S_{\geq 2}$, as we have defined and calculated it, differs from the definition of ΔS_{SS}. Similarly, the Ben-Naim ΔS_{PS} does not correspond to our ΔS_1.

As a result, whereas the Ben-Naim "solvent reorganization" cannot provide any net thermodynamic driving force, our solvent correlations, as measured by an MI expansion, are generally not compensated by ΔU_{SS} and therefore provide new insight, to our knowledge, into thermodynamic driving forces.

As the full ensemble of all potential molten-globule-like conformations is not sampled by our MD simulations, all differences of the free-energy contributions were calculated with respect to four ensemble members of the same molten-globule-like conformation. Particularly, the calculated protein entropy is therefore underestimated. For a rough estimate of the entropy error, we assume an effective number of 100–1000 metastable conformations (66,67) with similar conformational entropies. In this case, a configurational entropy contribution between $k_BT \log 100 = 11$ kJ mol^{-1} and $k_BT \log 1000 = 17$ kJ mol^{-1} would be missed, thereby not changing the budget qualitatively. As the first transition occurred after 1.5 μs (see Fig. S1), 1000 metastable states would correspond to an approximate but reasonable equilibration time of 1.5 ms.

With this caveat in mind, our calculations are in line with the known delicate balance between enthalpy and entropy, resulting a free-energy difference of (53 $\pm$ 84) kJ mol^{-1}, which corresponds to the enthalpy change due to just a few hydrogen bonds. The solvent entropy (free-energy contribution of $\sim$500 kJ mol^{-1}) was found to be one of the most important stabilizing contributions to the free-energy difference. Our study therefore also provides quantitative mechanistic insights into cold denaturation, driven by temperature-induced weakening of hydrophobic interactions (2). Indeed, the obtained mean entropy and enthalpy contributions would yield cold and heat denaturation temperatures of Crambin within the range of -42 to -22°C and 60 to 68°C, respectively, assuming protein-typical heat capacity differences ΔC_P between 5 and 15 kJ mol^{-1} K^{-1} (2,68) (see Supporting materials and methods). These temperature ranges agree with typical unfolding temperatures observed for globular proteins (2,6,7). The spatial resolution of the solvent free-energy terms identified favorable free-energy contributions due to solvated hydrophilic residues (charged or polar) as significantly larger than unfavorable contributions of solvated hydrophobic amino acids. The methodology might be relevant for computational in the context of computational protein design.

Because the local free-energy contributions around each residue also depend on the stereochemical environment formed by adjacent residues, the results presented here are specific to Crambin in the assessed native fold and the molten-globule-like configuration. Nevertheless, because Crambin exhibits a number of stereochemical motifs also present in most other globular proteins, it seems quite likely that the observed effects are, at least qualitatively, features of most globular proteins in general.

SUPPORTING MATERIAL

Supporting material can be found online at https://doi.org/10.1016/j.bpj.2021.05.019.

AUTHOR CONTRIBUTIONS

L.P.H. performed the research, analyzed data, and co-wrote the manuscript. H.G. designed the research and co-wrote the manuscript.

ACKNOWLEDGMENTS

L.P.H. thanks the International Max Planck Research School for Physics of Biological and Complex Systems for support through a PhD fellowship.

REFERENCES

1. Privalov, P. L., and N. N. Khechinashvili. 1974. A thermodynamic approach to the problem of stabilization of globular protein structure: a calorimetric study. *J. Mol. Biol.* 86:665–684.
2. Dias, C. L., T. Ala-Nissila, ..., M. Karttunen. 2010. The hydrophobic effect and its role in cold denaturation. *Cryobiology.* 60:91–99.
3. Lattman, E. E., and G. D. Rose. 1993. Protein folding–what's the question? *Proc. Natl. Acad. Sci. USA.* 90:439–441.
4. Agashe, V. R., and J. B. Udgaonkar. 1995. Thermodynamics of denaturation of barstar: evidence for cold denaturation and evaluation of the interaction with guanidine hydrochloride. *Biochemistry.* 34:3286–3299.
5. Wendler, K., J. Thar, ..., B. Kirchner. 2010. Estimating the hydrogen bond energy. *J. Phys. Chem. A.* 114:9529–9536.
6. Sanfelice, D., and P. A. Temussi. 2016. Cold denaturation as a tool to measure protein stability. *Biophys. Chem.* 208:4–8.
7. Kim, S. B., J. C. Palmer, and P. G. Debenedetti. 2016. Computational investigation of cold denaturation in the Trp-cage miniprotein. *Proc. Natl. Acad. Sci. USA.* 113:8991–8996.
8. Kauzmann, W. 1959. Some factors in the interpretation of protein denaturation. *Adv. Protein Chem.* 14:1–63.
9. Chandler, D. 2005. Interfaces and the driving force of hydrophobic assembly. *Nature.* 437:640–647.
10. Dill, K. A., T. M. Truskett, ..., B. Hribar-Lee. 2005. Modeling water, the hydrophobic effect, and ion solvation. *Annu. Rev. Biophys. Biomol. Struct.* 34:173–199.
11. Reinhard, F., and H. Grubmüller. 2007. Estimation of absolute solvent and solvation shell entropies via permutation reduction. *J. Chem. Phys.* 126:014102.
12. Reinhard, F., O. F. Lange, ..., H. Grubmüller. 2009. g_permute: permutation-reduced phase space density compaction. *Comput. Phys. Commun.* 180:455–458.
13. Heinz, L. P., and H. Grubmüller. 2020. Computing spatially resolved rotational hydration entropies from atomistic simulations. *J. Chem. Theory Comput.* 16:108–118.

14. Heinz, L. P., and H. Grubmüller. 2021. Per|Mut: spatially resolved hydration entropies from atomistic simulations. *J. Chem. Theory Comput.* 17:2090–2098.

15. Wallace, D. C. 1987. On the role of density fluctuations in the entropy of a fluid. *J. Chem. Phys.* 87:2282–2284.

16. Baranyai, A., and D. J. Evans. 1989. Direct entropy calculation from computer simulation of liquids. *Phys. Rev. A Gen. Phys.* 40:3817–3822.

17. Lazaridis, T. 1998. Inhomogeneous fluid approach to solvation thermodynamics. 1. Theory. *J. Phys. Chem. B.* 102:3531–3541.

18. Lazaridis, T. 1998. Inhomogeneous fluid approach to solvation thermodynamics. 2. Applications to simple fluids. *J. Phys. Chem. B.* 102:3542–3550.

19. Nguyen, C. N., T. K. Young, and M. K. Gilson. 2012. Grid inhomogeneous solvation theory: hydration structure and thermodynamics of the miniature receptor cucurbit[7]uril. *J. Chem. Phys.* 137:044101.

20. Nguyen, C. N., T. K. Young, and M. K. Gilson. 2012. Erratum: "Grid inhomogeneous solvation theory: hydration structure and thermodynamics of the miniature receptor cucurbit[7]uril" (J. Chem. Phys. 137, 044101 (2012)). *J. Chem. Phys.* 137:149901.

21. Nguyen, C. N., T. Kurtzman, and M. K. Gilson. 2016. Spatial decomposition of translational water-water correlation entropy in binding pockets. *J. Chem. Theory Comput.* 12:414–429.

22. Gerogiokas, G., G. Calabro, …, J. Michel. 2014. Prediction of small molecule hydration thermodynamics with grid cell theory. *J. Chem. Theory Comput.* 10:35–48.

23. Lin, S.-T., M. Blanco, and W. A. Goddard, III. 2003. The two-phase model for calculating thermodynamic properties of liquids from molecular dynamics: validation for the phase diagram of Lennard-Jones fluids. *J. Chem. Phys.* 119:11792–11805.

24. Lin, S.-T., P. K. Maiti, and W. A. Goddard, III. 2010. Two-phase thermodynamic model for efficient and accurate absolute entropy of water from molecular dynamics simulations. *J. Phys. Chem. B.* 114:8191–8198.

25. Persson, R. A. X., V. Pattni, …, M. Heyden. 2017. Signatures of solvation thermodynamics in spectra of intermolecular vibrations. *J. Chem. Theory Comput.* 13:4467–4481.

26. Berendsen, H. J., D. van der Spoel, and R. van Drunen. 1995. GROMACS: a message-passing parallel molecular dynamics implementation. *Comput. Phys. Commun.* 91:43–56.

27. Van Der Spoel, D., E. Lindahl, …, H. J. Berendsen. 2005. GROMACS: fast, flexible, and free. *J. Comput. Chem.* 26:1701–1718.

28. Hess, B., C. Kutzner, …, E. Lindahl. 2008. GROMACS 4: algorithms for highly efficient, load-balanced, and scalable molecular simulation. *J. Chem. Theory Comput.* 4:435–447.

29. Pronk, S., S. Páll, …, E. Lindahl. 2013. GROMACS 4.5: a high-throughput and highly parallel open source molecular simulation toolkit. *Bioinformatics.* 29:845–854.

30. Pall, S., M. J. Abraham, …, E. Lindahl. 2014. Tackling exascale software challenges in molecular dynamics simulations with GROMACS. *In* International Conference on Exascale Applications and Software. S. Markidis and E. Laure, eds. Springer, pp. 3–27.

31. MacKerell, A. D., Jr., D. Bashford, …, M. Karplus. 1998. All-atom empirical potential for molecular modeling and dynamics studies of proteins. *J. Phys. Chem. B.* 102:3586–3616.

32. Huang, J., and A. D. MacKerell, Jr. 2013. CHARMM36 all-atom additive protein force field: validation based on comparison to NMR data. *J. Comput. Chem.* 34:2135–2145.

33. Huang, J., S. Rauscher, …, A. D. MacKerell, Jr. 2017. CHARMM36m: an improved force field for folded and intrinsically disordered proteins. *Nat. Methods.* 14:71–73.

34. Jorgensen, W. L., J. Chandrasekhar, …, M. L. Klein. 1983. Comparison of simple potential functions for simulating liquid water. *J. Chem. Phys.* 79:926–935.

35. Bussi, G., D. Donadio, and M. Parrinello. 2007. Canonical sampling through velocity rescaling. *J. Chem. Phys.* 126:014101.

36. Berendsen, H. J., J. v. Postma, …, J. R. Haak. 1984. Molecular dynamics with coupling to an external bath. *J. Chem. Phys.* 81:3684–3690.

37. Andersen, H. C. 1980. Molecular dynamics simulations at constant pressure and/or temperature. *J. Chem. Phys.* 72:2384–2393.

38. Parrinello, M., and A. Rahman. 1981. Polymorphic transitions in single crystals: a new molecular dynamics method. *J. Appl. Phys.* 52:7182–7190.

39. Darden, T., D. York, and L. Pedersen. 1993. Particle mesh Ewald: an N log (N) method for Ewald sums in large systems. *J. Chem. Phys.* 98:10089–10092.

40. Jones, J. E. 1924. On the determination of molecular fields.—II. From the equation of state of a gas. *Proc. R. Soc. Lond. A.* 106:463–477.

41. Miyamoto, S., and P. A. Kollman. 1992. Settle: an analytical version of the SHAKE and RATTLE algorithm for rigid water models. *J. Comput. Chem.* 13:952–962.

42. Hess, B., H. Bekker, …, J. G. Fraaije. 1997. LINCS: a linear constraint solver for molecular simulations. *J. Comput. Chem.* 18:1463–1472.

43. Teeter, M. M., S. M. Roe, and N. H. Heo. 1993. Atomic resolution (0.83 A) crystal structure of the hydrophobic protein crambin at 130 K. *J. Mol. Biol.* 230:292–311.

44. Humphrey, W., A. Dalke, and K. Schulten. 1996. VMD: visual molecular dynamics. *J. Mol. Graph.* 14:33–38, 27–28.

45. Matsuda, H. 2000. Physical nature of higher-order mutual information: intrinsic correlations and frustration. *Phys. Rev. E Stat. Phys. Plasmas Fluids Relat. Interdiscip. Topics.* 62:3096–3102.

46. Hnizdo, V., E. Darian, …, H. Singh. 2007. Nearest-neighbor nonparametric method for estimating the configurational entropy of complex molecules. *J. Comput. Chem.* 28:655–668.

47. Hnizdo, V., J. Tan, …, M. K. Gilson. 2008. Efficient calculation of configurational entropy from molecular simulations by combining the mutual-information expansion and nearest-neighbor methods. *J. Comput. Chem.* 29:1605–1614.

48. Fengler, M. 2011. Estimating orientational water entropy at protein interfaces. Georg-August-Universität Göttingen, PhD thesis.

49. Schlitter, J. 1993. Estimation of absolute and relative entropies of macromolecules using the covariance matrix. *Chem. Phys. Lett.* 215:617–621.

50. Heyden, M. 2019. Disassembling solvation free energies into local contributions—toward a microscopic understanding of solvation processes. *Wiley Interdiscip. Rev. Comput. Mol. Sci.* 9:e1390.

51. Zhao, G., and E. London. 2006. An amino acid "transmembrane tendency" scale that approaches the theoretical limit to accuracy for prediction of transmembrane helices: relationship to biological hydrophobicity. *Protein Sci.* 15:1987–2001.

52. Ben-Naim, A. 1975. Hydrophobic interaction and structural changes in the solvent. *Biopolymers.* 14:1337–1355.

53. Ben-Naim, A., and Y. Marcus. 1984. Solvation thermodynamics of nonionic solutes. *J. Chem. Phys.* 81:2016–2027.

54. Yu, H.-A., and M. Karplus. 1988. A thermodynamic analysis of solvation. *J. Chem. Phys.* 89:2366–2379.

55. Tielrooij, K. J., S. T. van der Post, …, H. J. Bakker. 2011. Anisotropic water reorientation around ions. *J. Phys. Chem. B.* 115:12638–12647.

56. Lee, B. 1994. Enthalpy-entropy compensation in the thermodynamics of hydrophobicity. *Biophys. Chem.* 51:271–277; discussion 277–278.

57. Galamba, N. 2013. Water's structure around hydrophobic solutes and the iceberg model. *J. Phys. Chem. B.* 117:2153–2159.

58. Noskov, S. Y., G. Lamoureux, and B. Roux. 2005. Molecular dynamics study of hydration in ethanol-water mixtures using a polarizable force field. *J. Phys. Chem. B.* 109:6705–6713.

59. Denisov, V. P., K. Venu, …, B. Halle. 1997. Orientational disorder and entropy of water in protein cavities. *J. Phys. Chem. B.* 101:9380–9389.

60. Huggins, D. J. 2015. Quantifying the entropy of binding for water molecules in protein cavities by computing correlations. *Biophys. J.* 108:928–936.

61. von Domaros, M., D. Bratko, …, A. Luzar. 2019. Multifaceted water dynamics in spherical nanocages. *J. Phys. Chem. C.* 123:5989–5998.

62. Rhee, Y. M., and V. S. Pande. 2003. Multiplexed-replica exchange molecular dynamics method for protein folding simulation. *Biophys. J.* 84:775–786.

63. Sugita, Y., and Y. Okamoto. 1999. Replica-exchange molecular dynamics method for protein folding. *Chem. Phys. Lett.* 314:141–151.

64. García, A. E., and K. Y. Sanbonmatsu. 2001. Exploring the energy landscape of a β hairpin in explicit solvent. *Proteins.* 42:345–354.

65. Sanbonmatsu, K. Y., and A. E. García. 2002. Structure of Met-enkephalin in explicit aqueous solution using replica exchange molecular dynamics. *Proteins.* 46:225–234.

66. Noé, F., I. Horenko, …, J. C. Smith. 2007. Hierarchical analysis of conformational dynamics in biomolecules: transition networks of metastable states. *J. Chem. Phys.* 126:155102.

67. Noé, F., C. Schütte, …, T. R. Weikl. 2009. Constructing the equilibrium ensemble of folding pathways from short off-equilibrium simulations. *Proc. Natl. Acad. Sci. USA.* 106:19011–19016.

68. Robertson, A. D., and K. P. Murphy. 1997. Protein structure and the energetics of protein stability. *Chem. Rev.* 97:1251–1268.

Biophysical Journal, Volume *120*

Supplemental information

Spatially resolved free-energy contributions of native fold and molten-globule-like Crambin

Leonard P. Heinz and Helmut Grubmüller

Supplemental Information

Spatially resolved free-energy contributions of native fold and molten-globule-like Crambin

Leonard P. Heinz[1,*] and Helmut Grubmüller[1,*]

[1]Department of Theoretical and Computational Biophysics, Max-Planck Institute for Biophysical Chemistry, Göttingen, Germany

[*]Correspondence: lheinz@gwdg.de, hgrubmu@gwdg.de

UNRESTRAINED MD SIMULATION FOR THE PROTEIN ENTROPY CALCULATION

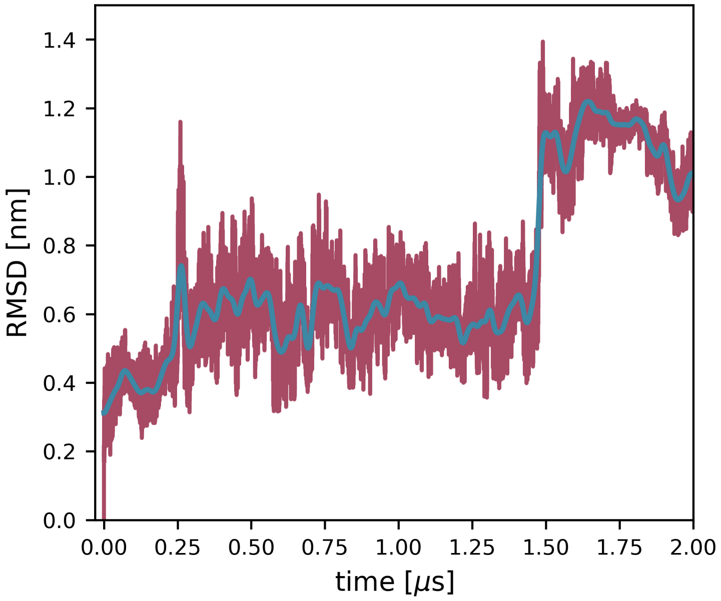

Figure S1: RMSD of the molten-globule-like conformation during an unrestrained MD simulation for the protein entropy calculation or Crambin. The 10 ns running average is shown in blue.

DECREASE OF WATER-WATER CORRELATIONS UPON BINDING TO A PROTEIN CHARGE

To show that the mutual information between a bound water molecule and a second-shell water molecule is smaller compared to bulk water, we considered two molecules with positions x and y.

In bulk, the molecules have single-body entropies $S(X)$, $S(Y)$ and the joint entropy $S(X,Y)$. Their mutual information (correlation) is $I(X,Y) = S(X) + S(Y) - S(X,Y)$.

Upon binding of the first molecule (X) to a protein charge, its entropy $\tilde{S}(X) < S(X)$ is reduced compared to bulk water

(see main text Fig. 2A). As shown by the distribution of $-T\Delta S_1$ in the main text Fig. 2A, the second-shell molecule is almost unaffected by the binding of X, i.e., its single-body entropy $\tilde{S}(Y) = S(Y)$ remains unchanged. We furthermore assume that the conditional entropy $\tilde{S}(X|Y) \leq S(X|Y)$ is also reduced compared to bulk water, as the mobility of X is hindered upon binding.

The mutual information

$$\begin{aligned}\tilde{I}(X,Y) &= \tilde{S}(X) + \tilde{S}(Y) - \tilde{S}(X,Y) \\ &= \tilde{S}(X) - \tilde{S}(X|Y) \\ &< S(X) - S(X|Y) = I(X,Y)\end{aligned}$$

is therefore smaller compared to bulk.

COMPARISON OF THE LOCAL CONVEXITY WITH THE ENTROPIC MULTI-BODY CONTRIBUTIONS

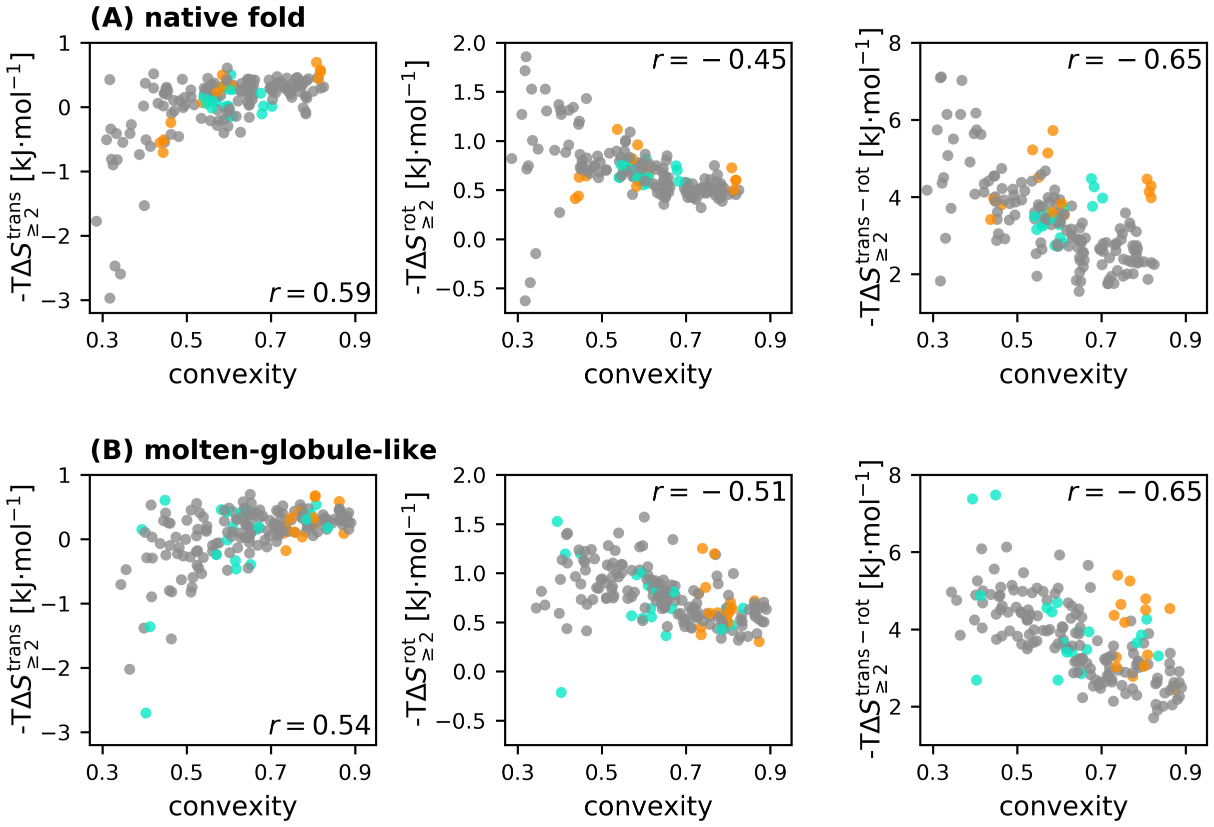

Figure S2: Dependence of the multi-body correlation contributions $-T\Delta S_{\geq 2}$ (relative to bulk water) on the local convexity for the native fold (A) and the molten-globule-like conformations (B). Charged amino acids are colored orange, polar amino acids are shown in cyan. Apolar amino acids are colored grey. Pearson correlation coefficients are stated in the corners of each plot.

ESTIMATION OF THE DENATURATION TEMPERATURES FROM THE SIMULATION RESULTS

The temperature dependence of the enthalpy difference between folded and unfolded states of a protein can be approximated as (1)

$$\Delta H(T) = \Delta H(T_c) + (T - T_c)\Delta C_P,$$

where T_c is the heat denaturation temperature and ΔC_P is the heat capacity difference between folded and unfolded states, which here is assumed to be constant. In the same manner, the entropy difference reads (1)

$$\Delta S(T) = \frac{\Delta H(T_c)}{T_c} + \Delta C_P \log\left(\frac{T}{T_c}\right).$$

Using the simulation results $\Delta H_{300} = \Delta H(300\,\mathrm{K}) \approx 228\,\mathrm{kJ{\cdot}mol^{-1}}$ and $-300\,\mathrm{K} \cdot \Delta S_{300} = -300\,\mathrm{K} \cdot \Delta S(300\,\mathrm{K}) \approx$

$-175\,\mathrm{kJ{\cdot}mol^{-1}}$, the above expressions can be rewritten as

$$\Delta H(T) = \Delta H_{300} + (T - 300\,\mathrm{K})\Delta C_P$$
$$\Delta S(T) = \Delta S_{300} + \Delta C_P \log\left(\frac{T}{300\,\mathrm{K}}\right).$$

Assuming typical values of ΔC_P between 5 and $15\,\mathrm{kJ{\cdot}mol^{-1}{\cdot}K^{-1}}$ (1, 2), the free-energy difference $\Delta G(T) = \Delta H(T) - T\Delta S(T)$ has a concave shape, as illustrated in Fig. S3.

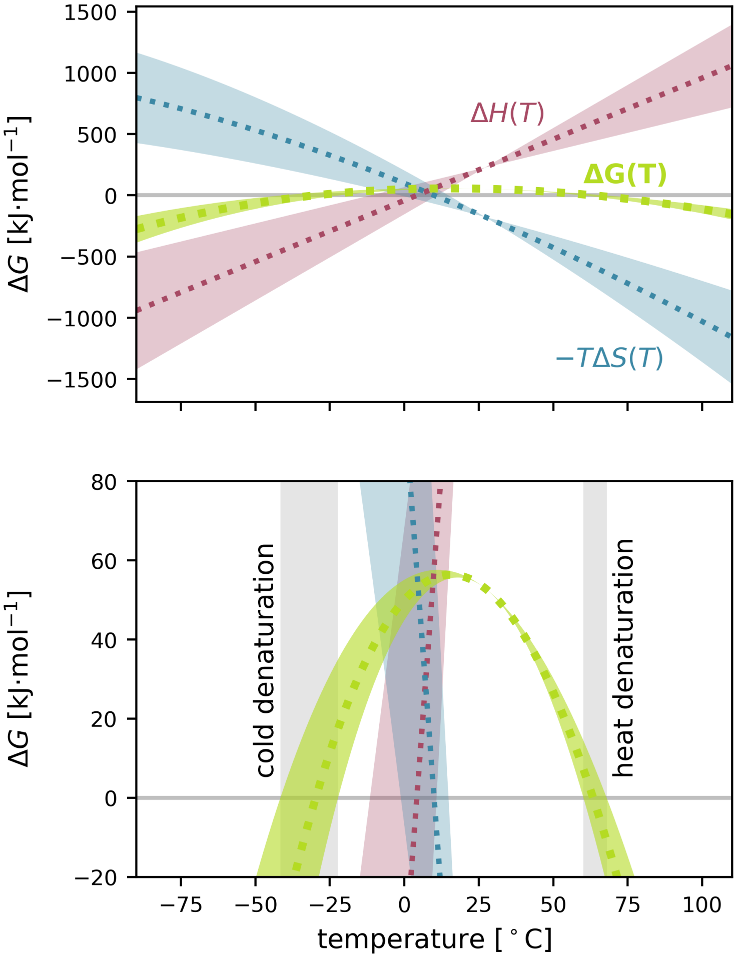

Figure S3: Temperature dependence of $\Delta H(T)$, $\Delta S(T)$, and $\Delta G(T)$ for ΔC_P between 5 and $15\,\mathrm{kJ{\cdot}mol^{-1}{\cdot}K^{-1}}$. The bottom panel is a zoomed-in version. Cold and heat denaturation temperature ranges are marked in grey.

The roots of $\Delta G(T)$, correspond to the cold (between $-42\,^\circ\mathrm{C}$ and $-22\,^\circ\mathrm{C}$) and heat denaturation (between $60\,^\circ\mathrm{C}$ and $68\,^\circ\mathrm{C}$) temperatures, respectively.

SPATIALLY RESOLVED THERMODYNAMIC QUANTITIES ON OFF-CENTER SLICES

The following figures show the spatially resolved solvent enthalpies, entropies, and free energies on slices across the three dimensional space, as visualized in Fig. 2 of the main text, but offset by 1.0, 0.5, −0.5, and −1.0 nm in depth relative to the protein center.

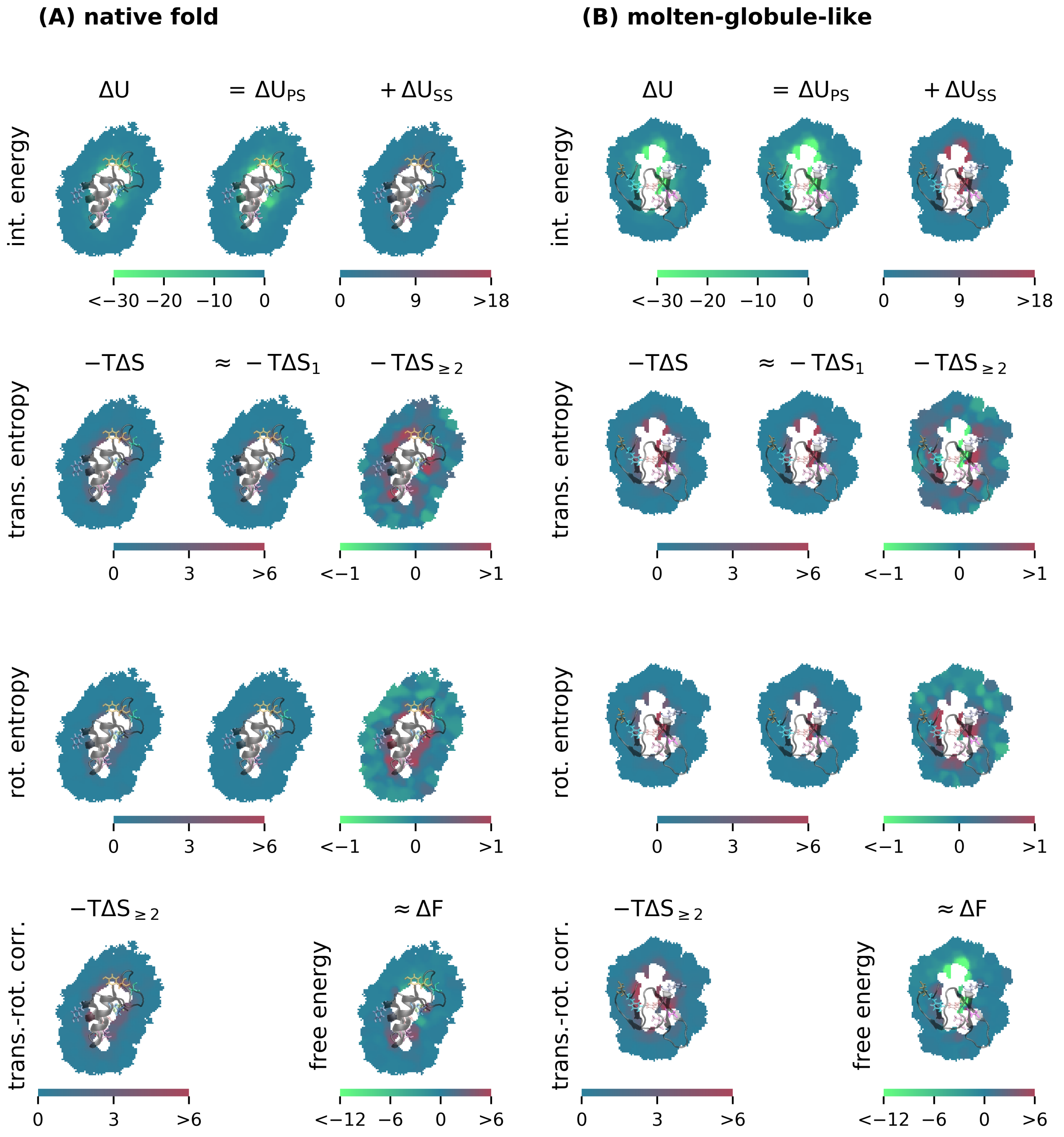

Figure S4: Spatially resolved solvent enthalpies, entropies, and free energies of Crambin in the native fold (A) and a molten-globule-like conformation (B), as shown in Fig. 2 of the main text. Visualization-slice across the three-dimensional space offset by $\approx 1.0\,\mathrm{nm}$ relative to the center of the protein.

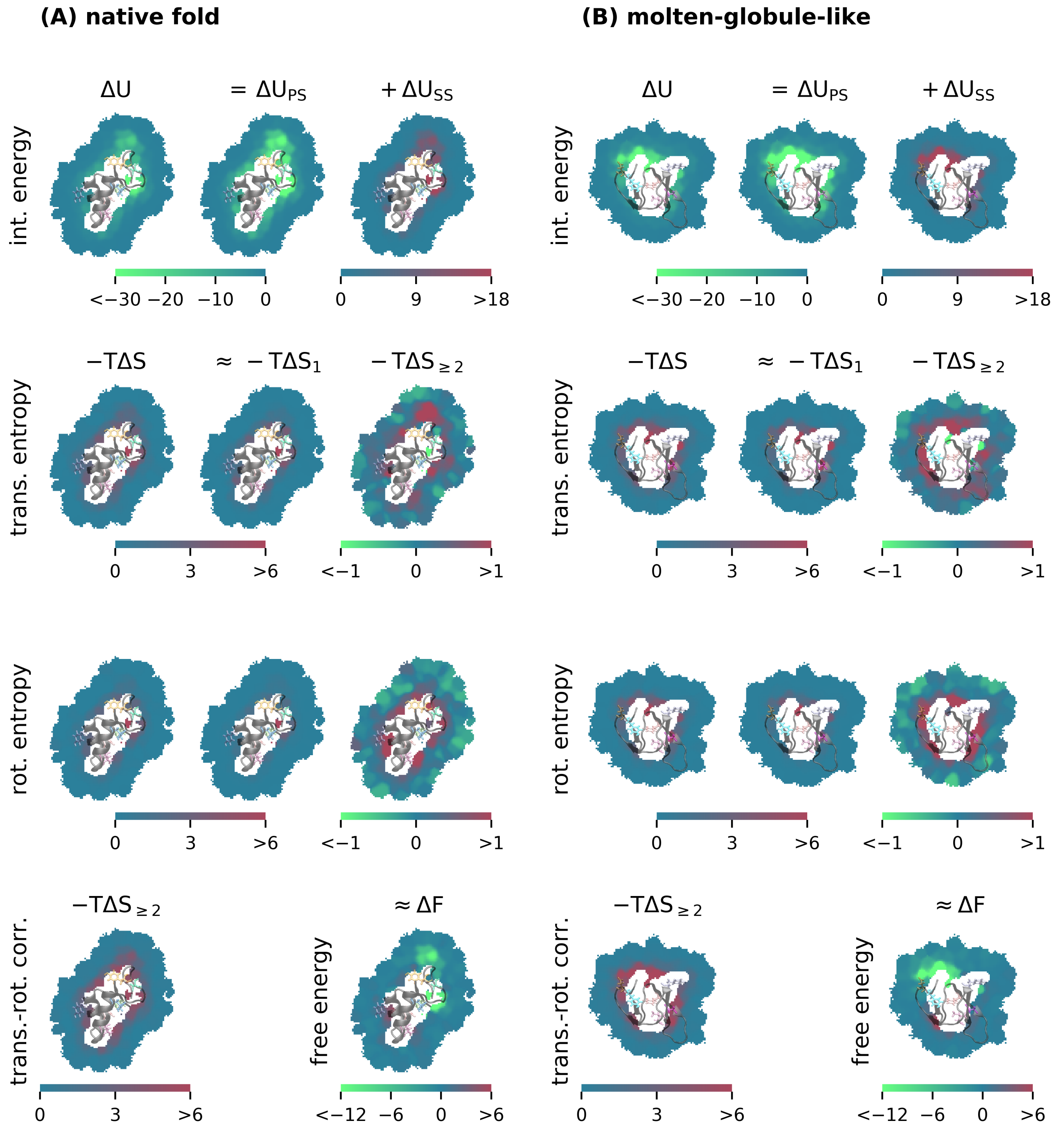

Figure S5: Spatially resolved solvent enthalpies, entropies, and free energies of Crambin in the native fold (A) and a molten-globule-like conformation (B), as shown in Fig. 2 of the main text. Visualization-slice across the three-dimensional space offset by ≈ 0.5 nm relative to the center of the protein.

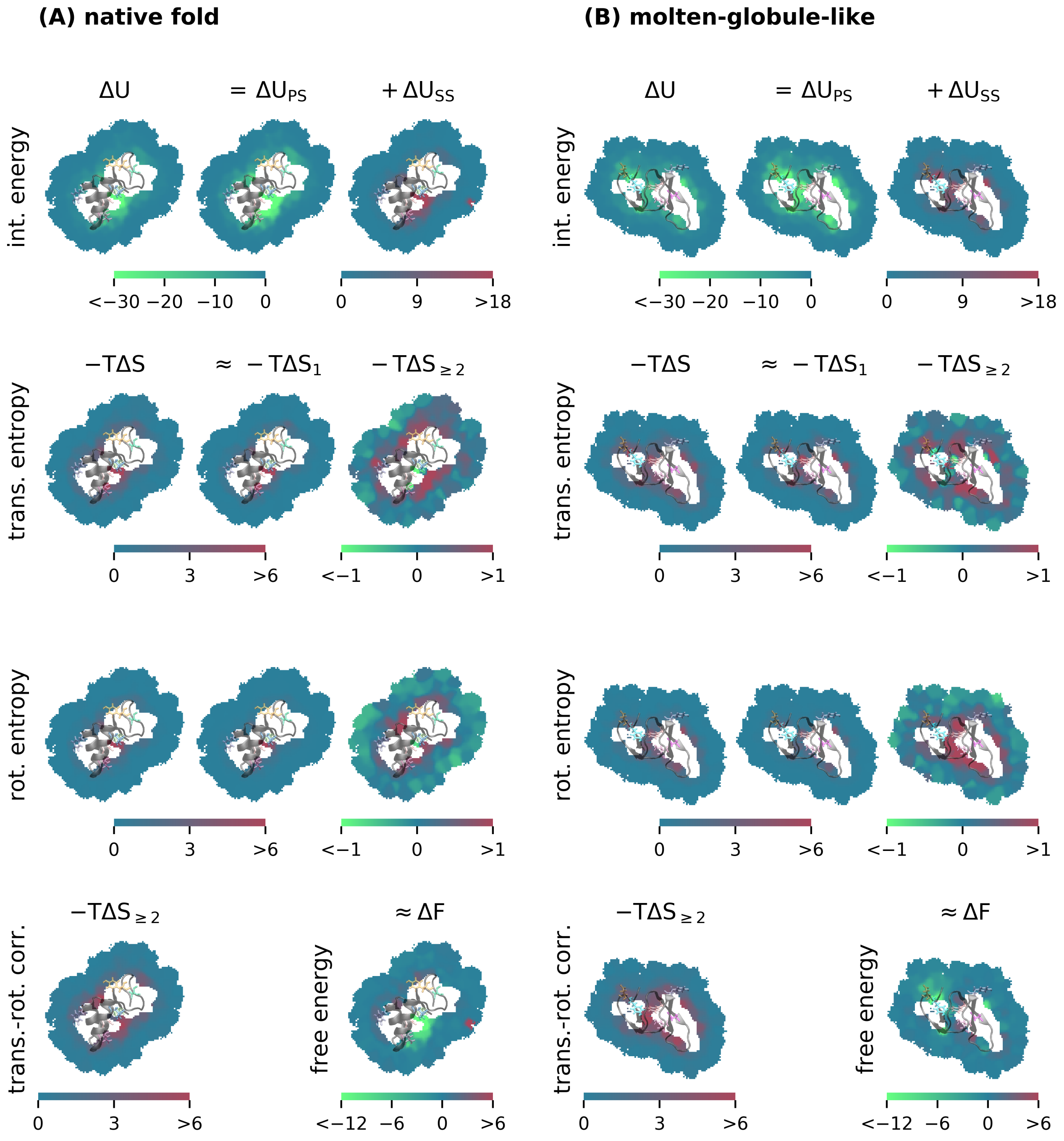

Figure S6: Spatially resolved solvent enthalpies, entropies, and free energies of Crambin in the native fold (A) and a molten-globule-like conformation (B), as shown in Fig. 2 of the main text. Visualization-slice across the three-dimensional space offset by ≈ -0.5 nm relative to the center of the protein.

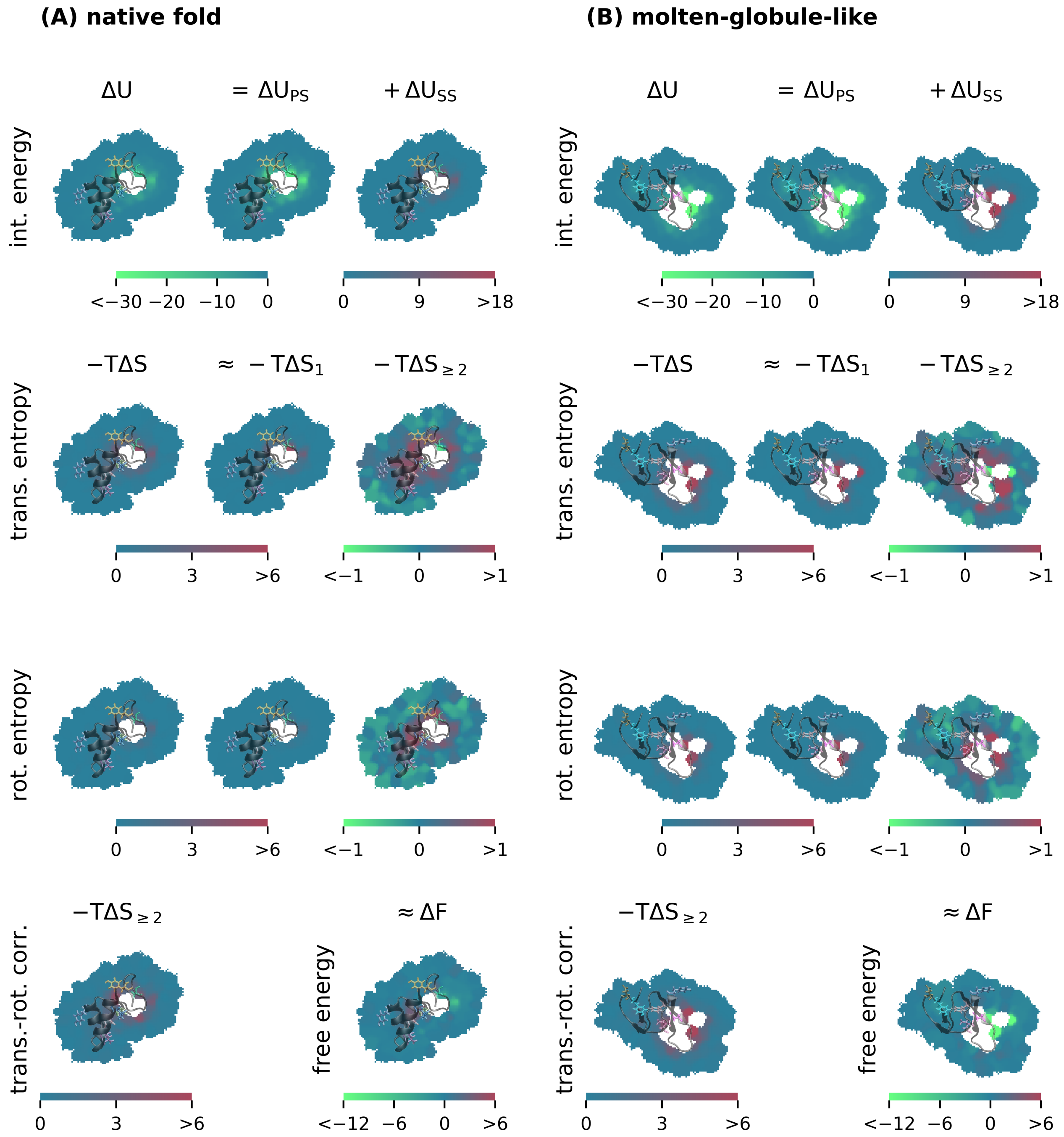

Figure S7: Spatially resolved solvent enthalpies, entropies, and free energies of Crambin in the native fold (A) and a molten-globule-like conformation (B), as shown in Fig. 2 of the main text. Visualization-slice across the three-dimensional space offset by $\approx -1.0\,\mathrm{nm}$ relative to the center of the protein.

REFERENCES

1. Dias, C. L., T. Ala-Nissila, J. Wong-ekkabut, I. Vattulainen, M. Grant, and M. Karttunen, 2010. The hydrophobic effect and its role in cold denaturation. *Cryobiology* 60:91–99.

2. Robertson, A. D., and K. P. Murphy, 1997. Protein structure and the energetics of protein stability. *Chemical reviews* 97:1251–1268.
